# Deeper insight into chronic kidney disease-related atherosclerosis: comparative proteomic studies of blood plasma using 2DE and mass spectrometry

**DOI:** 10.1186/s12967-014-0378-8

**Published:** 2015-01-27

**Authors:** Magdalena Luczak, Dorota Formanowicz, Łukasz Marczak, Elżbieta Pawliczak, Maria Wanic-Kossowska, Marek Figlerowicz, Maciej Stobiecki

**Affiliations:** Institute of Bioorganic Chemistry, Polish Academy of Sciences, Noskowskiego 12/14, 61-704 Poznan, Poland; Department of Clinical Biochemistry, Poznan University of Medical Sciences, Grunwaldzka 6, 60-780 Poznan, Poland; Department of Nephrology, Transplantology and Internal Medicine, Poznan University of Medical Sciences, Przybyszewskiego 49, 60-355 Poznan, Poland; Institute of Computing Science, Poznan University of Technology, Piotrowo 2, 60-965 Poznan, Poland; Institute of Chemical Technology and Engineering, Poznan University of Technology, Piotrowo 3A, 60-965 Poznan, Poland

**Keywords:** Chronic kidney disease, Atherosclerosis, Cardiovascular disease, Proteome profiling, Mass spectrometry

## Abstract

**Background:**

Atherosclerosis is a major cause of cardiac events and mortality in patients suffering from chronic kidney disease (CKD). Moreover, the risk of cardiovascular disease (CVD) development in patients with CKD increases as kidney function declines. Although the close connection between atherosclerosis and kidney dysfunction is undeniable, particular risk factors and specific mechanisms that promote CVD in patients with CKD remain unclear. To gain insight into better recognition of the mechanisms of accelerated atherosclerosis in patients with CKD, we performed a comparative proteomic analysis of blood plasma from patients in various stages of CKD and thus distinct progression of atherosclerosis (n = 90), patients with advanced CVD and normal renal function (n = 30) and healthy volunteers (n = 30).

**Methods:**

Plasma samples were depleted using affinity chromatography and divided into three fractions: high-abundant, low-abundant and low-molecular weight proteins. The first two fractions were analyzed by two-dimensional gel electrophoresis and mass spectrometry, the last one has been subjected to direct MS/MS analysis. A proteomic profiles for high-abundant, low-abundant and low-molecular weight proteins fractions were obtained. Differential accumulated proteins were confirmed by selected reaction monitoring analysis (SRM). The Gene Ontology (GO) function and the interaction networks of differentially expressed proteins were then analyzed.

**Results:**

Forty-nine proteins (13 high- and 36 low-molecular mass) showed differences in accumulation levels. For eleven of them differential expression were confirmed by selected reaction monitoring analysis. Bioinformatic analysis showed that identified differential proteins were related to three different processes: the blood coagulation cascade, the transport, binding and metabolism of lipoproteins and inflammatory processes.

**Conclusions:**

Obtained data provide an additional line of evidence that different molecular mechanisms are involved in the development of CKD- and CVD-related atherosclerosis. The abundance of some anti-atherogenic factors revealed in patients with CKD suggests that these factors are not associated with the reduction of atherosclerosis progression in CKD that is typically observed in “classical” CVD. Moreover, obtained data also suggest that mechanism of CVD acceleration may be different in initial and advanced stages of CKD. Undoubtedly, in advanced stages of CKD inflammation is highly pronounced.

**Electronic supplementary material:**

The online version of this article (doi:10.1186/s12967-014-0378-8) contains supplementary material, which is available to authorized users.

## Introduction

Chronic kidney disease (CKD) is defined as impaired kidney function that is characterized by a progressive decrease in the glomerular filtration rate (GFR) or an increase in proteinuria over a period of at least 3 months [1]. Guidelines from the Kidney Disease Outcomes Quality Initiative (K/DOQI) [2] place patients with CKD into one of five stages, based on their estimated GFRs (eGFRs). The 1^st^ stage of CKD (CKD1) is the mildest one, and although eGFRs are similar to those observed in healthy people (eGFR above 90 mL/min/1.73 m^2^), urine findings or structural abnormalities suggest CKD. In the 2^nd^ stage of CKD (CKD2), a mild reduction in the GFR (eGFR between 89 and 60 mL/min/1.73 m^2^) is observed. In the 3^rd^ and 4^th^ stages of CKD, moderate and severe kidney damage occur (CKD3: GFR 30–59 mL/min/1.73 m^2^; CKD4: 15–29 mL/min/1.73 m^2^). The 5^th^ stage of CKD (CKD5) is the most advanced stage and is indicative of kidney failure (GFR <15 mL/min/1.73 m^2^).

Atherosclerosis is one of the most serious and frequent complications that occurs in patients suffering from CKD and is a major cause of mortality in this group of patients [3]. The risk of developing cardiovascular disease (CVD) can be predicted from the GFR, and increases as the GFR declines [4,5]. Additionally, CKD patients with GFR ≤60 mL/min/1.73 m^2^ are considered to have an elevated risk of cardiovascular events among them heart failure, ischemic heart disease, myocardial infarction and stroke [6]. Many studies in various populations have reported that a decreased GFR and increased albuminuria are associated with CVD in graded association. Cardiovascular mortality is approximately two times higher in patients with stage 3 CKD and three times higher in patients with stage 4 CKD than in individuals with normal kidney function [3]. Therefore, although the close connection between kidney dysfunction and atherosclerosis is indisputable, the frequency and severity of cardiovascular complications is disproportionate to the underlying risk factor profile.

In recent years, considerable research efforts have been directed toward identifying the mechanism of accelerated development of CVD in patients with CKD. Traditional risk factors that support the development of classical CVD (i.e., hypertension, hypercholesterolemia, obesity and hyperhomocysteinemia) alone cannot explain the high incidence of CVD in patients with CKD. Several other risk factors may be more important, including inflammation, endothelial dysfunction, oxidative stress, vascular calcification and volume overload. The close relationship between CVD and CKD is most likely due to the co-existence of both traditional and novel (non-traditional) cardiovascular risk factors. The combination of these factors may lead to surprising results. For example, the concentration of serum cholesterol has a widely recognized impact on atherogenesis in the general population. Paradoxically, the significance of this factor in the pathogenesis of CKD-related atherosclerosis (CKDA) is not so obvious, particularly in the advanced stages of CKD, wherein high serum cholesterol levels seem to protect against cardiovascular death. This observation, together with other similar findings, has led to the term ‘reverse epidemiology’ [7,8].

Thus, the exact participation and association of particular risk factors and specific mechanisms that promote CVD in patients with CKD remain unclear. To better understand these mechanisms, we have attempted to determine the changes induced in the proteome of blood plasma from patients with different stages of CKD and CVD. In our earlier studies we performed 2DE/MS analysis of raw plasma samples without depletion of high abundant proteins [9]. In the present work, we sought to expand the catalog of differentially expressed plasma proteins related to CKD and CKDA. Thus, we analyzed three different fractions of plasma proteins: high-abundance proteins (HAPs), low-abundance proteins (LAPs) and low-molecular-weight proteins (LMWPs, that is, below 5 kDa) in patients with diagnosed CKD and CVD as well as in healthy volunteers (HVs). All of the studied CKD patients revealed presence of atherosclerosis on different progression stage. All CVD patients showed advanced cardiovascular disease symptoms and had none clinical symptoms of renal dysfunction. All plasma protein fractions were compared to identify differentially accumulated proteins.

## Materials and methods

### Subjects and samples

The study protocol conforms to the ethical guidelines of the World Medical Association Declaration of Helsinki. Before the project commenced, appropriate approval was obtained from the Bioethical Commission of the Karol Marcinkowski University of Medical Sciences (no. 14/07 04.01.2007). All participating individuals provided signed informed consent for treatment and study. Characteristics of the studied population are shown in Table 1. Study involved 150 persons divided into five equal groups. The patients were matched for age and gender. The majority of them were patients with CKD (90 persons) who were treated in the Department of Nephrology, Transplantology and Internal Medicine at Poznan University of Medical Sciences. Based on the National Kidney Foundation Kidney Disease Outcomes Quality Initiative guidelines [2], the examined CKD patients were divided into three groups according to their eGFR, which was calculated by the formula developed by Levey et al. [10]. The first group, CKD1-2, contained patients in the initial stages of CKD (the 1^st^ and 2^nd^ stages of CKD, with eGFR = 90–60 mL/min/1.73 m^2^). The second group, CKD3-4, included pre-dialyzed patients in the 3^rd^ and 4^th^ stages of CKD, with eGFR = 59–15 mL/min/1.73 m^2^. The third group, CKD5, comprised patients with eGFR <15 mL/min/1.73 m^2^ who were hemodialyzed for 39.6 ± 9.5 months (mean ± SD) with prescriptions of 4.5-5.5 h/session, three times per week. CKD patients varied in progression of atherosclerosis and percentage of cardiovascular events. CKD1-2 showed the first symptoms of hypertension or ischemic heart disease. In more advanced stages of CKD mild and severe cardiovascular disease symptoms were observed. Fifty-nine percent of CKD5 patients have a history of myocardial infarction or stroke (Table 1).

**Table 1:** **Demographic data and clinical characteristics of the study population (n = 150)**

	**CKD1-2**	**CKD 3-4**	**CKD5**	**CVD**	**HV**	***p***
**Age [years]**	61.5 +/− 9.15	59.5+/− 6.3	60.1+/−10.1	61 +/− 7.07	60.5 +/− 10.1	NS
**Males/females**	17/13	17/13	17/13	17/13	17/13	NS
**eGFR [ml/min/1.73 m2]**	77.04 +/− 22.9	19.1 +/− 8.0	5.75 +/− 7.1	92.7 +/− 21.1	123.6 +/− 17.6	0.000
**BMI [kg/m2]**	27.11+/− 4.21	28.31+/− 1.21	25.74 +/− 3.42	28.98 +/− 3.21	24.98 +/− 2.87	0.000
**Arterial hypertension**	100%	100%	100%	100%	0%	0.000
**History of myocardial infarction/stroke**	15%	21%	59%	68%	0%	0.000
**Systolic BP [mmHg]**	124.76 +/− 13.21	138.21+/−7.26	134.42 +/− 26.78	115.28 +/− 12.23	124.32 +/− 12.31	0.002
**Diastolic BP [mmHg]**	76.27 +/− 11.12	89.12 +/− 74.23	77.13 +/− 19.21	71.21 +/− 11.23	68.34 +/− 14.21	0.021
**Statin treatment**	61%	49%	68%	92%	0%	0.000
**IACE treatment**	84%	48%	60%	76%	0%	0.000
**Total cholesterol [mg/dL]**	229.06 +/− 51.12	184.33 +/− 28.61	179.53 +/− 38.39	193.22 +/− 38.14	185.11 +/− 29.12	0.031
**HDL cholesterol [mg/dL]**	55.1 +/− 10.15	58.4 +/− 7.64	45.18 +/− 23.076	46.58 +/− 15.12	70.11 +/− 5.14	0.000
**LDL cholesterol [mg/dL]**	169.66 +/− 41.25	120.06 +/− 17.11	103.24 +/− 40.05	114.65 +/− 34.16	91.92 +/− 29.14	0.000
**Triglycerides [mg/dL]**	170.41 +/− 68.16	117.24 +/− 21.18	133.35 +/− 50.83	139.68 +/− 60.51	121.2 +/− 34.01	0.042
**hsCRP [mg/L]**	1.62 +/− 0.36	9.19 +/− 2.15	12.32 +/− 18.05	5.88 +/− 1.14	1.09 +/− 0.15	0.001
**CIMT [mm]**	0.71 +/− 0.21	0.83 +/− 024	0.92 +/− 0.42	0.72 +/− 0.32	0.45 +/− 0.28	0.000
**Diagnosis of CKD [number of patients]**						
Hypertensive nephropathy	10	11	12			
Chronic glomerulonephritis	7	7	7			
Chronic interstitial nephritis	9	6	6			
Polycystic kidney disease	0	1	2			
Others or unknown	4	5	3			

No. (%) or mean value ± SD. *p* < 0.05 was considered as statistically significant, NS - indicates not significant differences between all the studied groups. eGFR - estimated glomerular filtration rate, BMI - body mass index, BP - blood pressure, IACE - angiotensin-converting enzyme inhibitors, HDL cholesterol - high density lipoprotein cholesterol, LDL cholesterol - low density lipoprotein cholesterol, hsCRP - high-sensitivity C-reactive protein , CIMT - carotid intima media thickness.

A fourth group (called CVD) included 30 patients with a history and symptoms of the atherosclerotic occlusive disease, admitted for angiography to the Department of Internal Medicine, Division of Cardiac Intensive Care in Poznan University of Medical Sciences. All CVD patients have at least one artery stenosis causing lumen reduction of at least 50%. Sixty-eight percent of CVD patients revealed history of myocardial infarction or stroke. No subjects from the CVD group had any clinical symptoms of renal dysfunction.

A fifth control group (HV) contained 30 healthy volunteers. Persons with diabetes mellitus, acute inflammatory processes and malignant tumors either presently or within the past 10 years were excluded from the study.

All of the studied subjects were diagnosed with atherosclerosis on the basis of their medical history (history of myocardial infarction or/and ischemic stroke), their lipids metabolism parameters, systolic and diastolic blood pressure values and carotid intima media thickness (CIMT). For all CKD and CVD patients, blood samples were collected at the same time that standard monitoring blood tests were performed. In the case of hemodialyzed patients, blood samples were always drawn before the second hemodialysis session of the week. Peripheral blood was collected into a closed monovette system containing EDTA and was centrifuged immediately at 1,000 *g* for 15 min. The obtained supernatants were then centrifuged at 16,000 *g* for 15 min at 4°C and frozen at −80°C.

### Isolation of LAPs, HAPs and LMWPs from plasma samples

Immunoaffinity depletion was used to isolate LAPs, HAPs and LMWPs. Individual plasma samples were processed to decrease plasma complexity by depletion of highly abundant proteins with a MARS-Hu7 affinity column (Agilent Technologies, USA). The MARS-Hu7 spin column removed the 7 most abundant plasma proteins (human albumin, IgG, α1-antitrypsin, IgA, transferrin, HP and fibrinogen), which constitute approximately 90% of the plasma proteome. Human plasma (20 μL) from the patient and HV group was diluted to 400 μL with “Buffer A” (Agilent Technologies), centrifuged for 1 min through a 0.22 μm spin filter tube (Agilent Technologies, USA) at 14,000 *g* and then prepared according to the manufacturer’s instructions in two cycles. Aliquots of the flow-through fractions containing LAPs as well as the bound fractions with HAPs were desalted by buffer exchange run three times using centrifugal filter devices with a 5-kDa cutoff (Amicon Ultra, Millipore). The flow-through after filtration of the LAP fraction with 5-kDa filters was evaporated on a SpeedVac and then directly analyzed by MALDI-TOF/TOF as LMWPs. Samples were stored at −80°C prior to analysis, and the protein concentration was measured using a commercial 2-D Quant kit (GE Healthcare).

### 2-D electrophoresis

A total of 100 μg of the LAP fractions deriving from individual samples was separated using 7-cm IPG strips (pH 4–7, GE Healthcare) in four repetitions. 650 μg of the HAP fractions was separated using 24-cm IPG strips (pH 4–7) in at least three repetitions. Strips were actively rehydrated overnight in IEF buffer containing plasma proteins. The strips were subjected to IEF on IPGphor III (GE Healthcare) using a ramping voltage of 50–8,000 V to a final voltage of 75,000 Vh for 24-cm IPG strips and 50–5,000 V to 18,000 Vh for 7-cm strips. Reduction, alkylation and separation in the second dimension were performed as previously described [9,11]. After electrophoresis, gels were stained with Blue Silver overnight [12] and scanned using the LabScan program with a Umax scanner (GE Healthcare).

The images were analyzed using the Image Master Platinum software, version 6.0 (GE Healthcare). In total approximately 1,000 obtained images were analyzed. Spots were detected automatically without filtering. Gel patterns were automatically matched together between classes. In addition, all individually matched spots were validated manually to ensure that spot matching was correct. The relative abundance of each spot (%vol) was calculated as its volume divided by the total volume of matched spots. 2DE reproducibility was assessed by scatter plotting and correlation coefficient determination based on %vol parameter. Images showing the correlation coefficient below 0.7 were rejected from analysis. The final datasets consisted of 443 and 332 images for LAP and HAP fraction, respectively. Average relative abundance of protein spot for each group was calculated for all obtained %vol (all samples in all repetitions in group). To find differentially expressed proteins, gap and ratio measures were taken into account. For differentiating proteins threshold 1.6 was selected but only with restrictive *p* value below 0.001. However only proteins that were validated later with SRM analysis with a threshold greater than 1.5 were accepted as differential accumulating proteins. Protein spots were manually excised from gels, transferred to Eppendorf tubes and stored at −80°C prior to MS analysis.

### In-gel digestion

Individual protein spots were cut into small pieces, rinsed twice in 100 μL of washing buffer (50 mM NH_4_HCO_3_/100% ACN (vol. 1:1)) for 15 min and dehydrated in 100% ACN. The dried gel spots were rehydrated by the addition of 10 μL of digestion buffer (25 mM ammonium bicarbonate and 0.2 μg of sequencing-grade trypsin (Promega)). Digestion was performed overnight at 37°C. Peptides were extracted with 10% ACN.

### Mass spectrometry (MS)

#### Protein identification

Digested proteins were identified using a MALDI-TOF/TOF mass spectrometer. The MALDI spectra were acquired on an UltrafleXtreme (Bruker Daltonics, Germany) mass spectrometer operated in reflector mode using delayed ion extraction. Positively charged ions in the m/z range of 800–3500 were analyzed. For each sample, 0.5 μL was co-crystallized with CHCA matrix and spotted directly onto the MALDI AnchorChip target (Bruker Daltonics, Germany). The MS spectra were externally calibrated using the Peptide Calibration Standard mixture (Bruker Daltonics, Germany). Flex control v. 3.3 was used for the acquisition of spectra, and all further data processing was performed using Flex analysis v. 3.3. The spectrometric analysis was performed in an automatic dependent mode under the control of BioTools 3.2 and WARP-LC 1.3 software. Non-redundant precursor peptides were selected for MS/MS with a signal-to-noise threshold of 12. A maximum of 20 precursor ions per spot were chosen for MS/MS analysis. Monoisotopic peptide masses were assigned and used for databases research. Protein database searches using combined PMF and MS/MS datasets were performed via BioTools 3.2 (Bruker Daltonics, Germany). Proteins were identified using the Mascot (Matrix Science, London, UK) program against the SwissProt (2014; 545 388 sequences in total) and NCBI (2011; 13 366 630 sequences in total) databases. The false discovery rate (FDR) for peptide identification was 0.05 in all analyses. The protein search was performed using the following search parameters: precursor-ion mass tolerance, +/−0.1 Da; fragment-ion mass tolerance, +/−0.4 Da and cysteine-treated with iodoacetamide to form carbamidomethyl-cysteine and oxidized methionine. Trypsin was set as the enzyme, with a maximum of one missed cleavage. Proteins were identified on the basis of at least two unique peptides with peptide score higher than 40 (*p* < 0.05).

#### Analysis of LMWPs

Low-molecular proteins obtained from individual plasma samples after centrifugal ultrafiltration with a 5-kDa filter were analyzed directly by MS without electrophoretic separation. Samples that were not trypsin digested were co-crystallized with sinapinic acid matrix, spotted on a MALDI AnchorChip target and analyzed in the m/z range 2,000-12,000 Da. All samples were analyzed in five technical replications. For data validation, external calibration was performed with a standard mixture of proteins (Bruker Daltonics, Germany). Visualization and analysis of the obtained spectra as well as principal component analysis (PCA) were performed with ClinProTools 2.2 (Bruker Daltonics). Differential components with fold change >2 and *p* values below 0.01 were considered as statistically significant. Differential LMWPs were identified using TOF/TOF fragmentation. MS/MS data were searched against the Mascot with non-enzyme settings.

#### Validation of potential biomarkers by SRM

Proteins that were identified as differentially expressed were validated by SRM analysis. Two reaction monitoring transitions were determined empirically for all proteins. They were calculated based on the analysis of fragmentation spectra obtained after in-gel digestion of proteins that were identified as differentially expressed. The peptides and transitions selected for SRM analysis are presented in Additional file 1. Transitions for each peptide were analyzed in separate MS/MS experiments. All peptide sequences were analyzed with NCBI BLAST using non-redundant protein sequences database and blastp algorithm to ensure that the sequences were unique. Then, all transitions were tested using in-solution digested plasma proteins. SRM analyses were conducted on pulled of five plasma samples from each group. The plasma samples deriving from five individuals were diluted with Milli-Q water, aliquoted into 10 μg samples and pooled. The pooling strategy in each experimental group was as follows: 1^st^ SRM run consist samples 1 to 5; 2^nd^ run: 6 to 10; 3^rd^ run: 11 to 15; 4^th^ run: 16 to 20, 5^th^ run: 21 to 25 and 6^th^ run: 26 to 30. This scheme was repeated for each experimental group. Four separate aliquots of pooled plasma samples were processed as technical replicates. Therefore the final datasets consisted of 120 SRM analyses. We did not pulled all the samples in one set to minimize the loss of information about the biological variation. Therefore, this experimental design consists of a mix of biological and technical replicates. One microliter of prepared sample was mixed with 50 mM ammonium bicarbonate (pH 8.0) to a final volume of 60 μL. Then, the samples were reduced in the presence of 50 mM NH_4_HCO_3_ with 5.6 mM DTT for 5 min at 95°C and alkylated with 5 mM iodoacetamide for 20 min in the dark at RT. The proteins were digested using 0.2 μg of sequencing-grade trypsin overnight at 37°C. The obtained mixtures of peptides were analyzed in an ESI-Ion Trap (Amazon SL, Bruker Daltonics) mass spectrometer coupled with a UPLC system (nanoAQUITY, Waters). The effluent from the nanoLC column, which is an RP C18 pre-column (nanoACQUITY) connected to a 25-cm-long, 75-μm-i.d. RP C18 column (nanoAQUITY), was directly introduced into the Ion Trap in positive electrospray ionization (ESI) mode. Samples were eluted from the column with a gradient of 4 to 60% ACN in 140 min (solvent A, 0.1% formic acid; solvent B, 99.9% ACN and 0.1% formic acid) at a flow rate of 0.3 μL/min. Ion trap charge control (ICC) was used to control ion accumulation in the trap. For precursor ion isolation, a 3-Da window was set up, and the precursor fragmentation amplitude was set to 1.0. Acquisitions were run under the control of Trap Control 7.1 software (Bruker Daltonics). All SRM data were processed using Data Analysis 4.0 software (Bruker Daltonics). The relative quantification values of each target peptide were determined by calculating the average ratios of peak areas corresponding to the analyzed peptides. All data were manually inspected to ensure correct peak detection and accurate integration.

### Statistical analysis

Clinical data: for multiple comparisons of the normally distributed variables the one-way analysis of variance (ANOVA) and the Kruskal - Wallis test have been performed. For categorical comparisons chi square test was performed. *P* values < 0.05 were considered statistically significant.

Proteomic data: in 2DE experiments, relative abundance (%vol) of all matched differential spots obtained for all individual samples in all technical repetitions were analyzed. Proteins with fold change at least 1.6 were statistically analyzed. For SRM and LMWP analysis, peak areas corresponding to the analyzed peptides in all runs were analyzed. All obtained data were subjected to a Shapiro-Wilk normality test to check the normal distribution of the analyzed population. Mann–Whitney U test was performed to compare protein accumulation between two particular groups. For multiple comparisons the one-way analysis of variance (ANOVA) with a Bonferroni correction for multiple testing was performed. *P* values < 0.001 and < 0.01 were considered as statistically significant for 2DE and SRM/LMWPs data, respectively. For obtained results regression and correlation analysis were also performed. All statistical analyses were performed using Statistica v. 10.0 software.

### Bioinformatics analysis

A protein-protein interaction analysis of the identified proteins was performed using STRING (Search Tool for the Retrieval of Interacting Genes) v. 8.3 (http://string.embl.de/) [13] with the following analysis parameters: species—Homo sapiens, confidence level—0.4 and active prediction methods—all. STRING is a freely available database that relies on known and predicted protein interactions and that quantitatively integrates interaction data from high-throughput experiments, genomic context, co-expression and other literature.

To classify proteins by gene ontology (GO) molecular function, biological processes and pathway terms both Panther (www.pantherdb.org) [14,15] and the ProteinScape software (Bruker Daltonics) were used. The complete dataset containing all protein identifiers as well as only differential proteins (>1.6-fold change and *p* value < 0.001 for 2DE; >2-fold change and *p* value < 0.01 for LMWP) were uploaded into the applications. The gene symbols of the differentially expressed proteins and accumulation levels were entered in Panther to run the enrichment test to evaluate likelihood of the numerical values of genes associated with a particular ontology term. *P*-values were calculated using the Mann–Whitney test with Bonferroni correction for multiple testing with p < 0.05 indicating statistical significance. The *p*-value for a given function was calculated by considering the number of differentially expressed proteins that participate in given function and the total number of molecules that are known to be associated with that function in Panther. The *p*-value becomes more significant where a greater number of differentially expressed proteins are involved.

## Results

To increase our knowledge of the mechanisms underlying the development of CKDA, we identified the proteins that are differentially accumulated in the plasma of patients with different stages of CKD, patients with CVD (and normal renal function) and HVs. The comparative proteomic analyses were performed between HVs and each of the groups of CKD patients, between CKD and CVD patients and between neighboring groups of CKD patients. In our studies, we focus on three different fractions of plasma proteins: HAPs, LAPs and LMWPs.

### Qualitative analysis of immunoaffinity depleted plasma proteins

**Figure 1:**
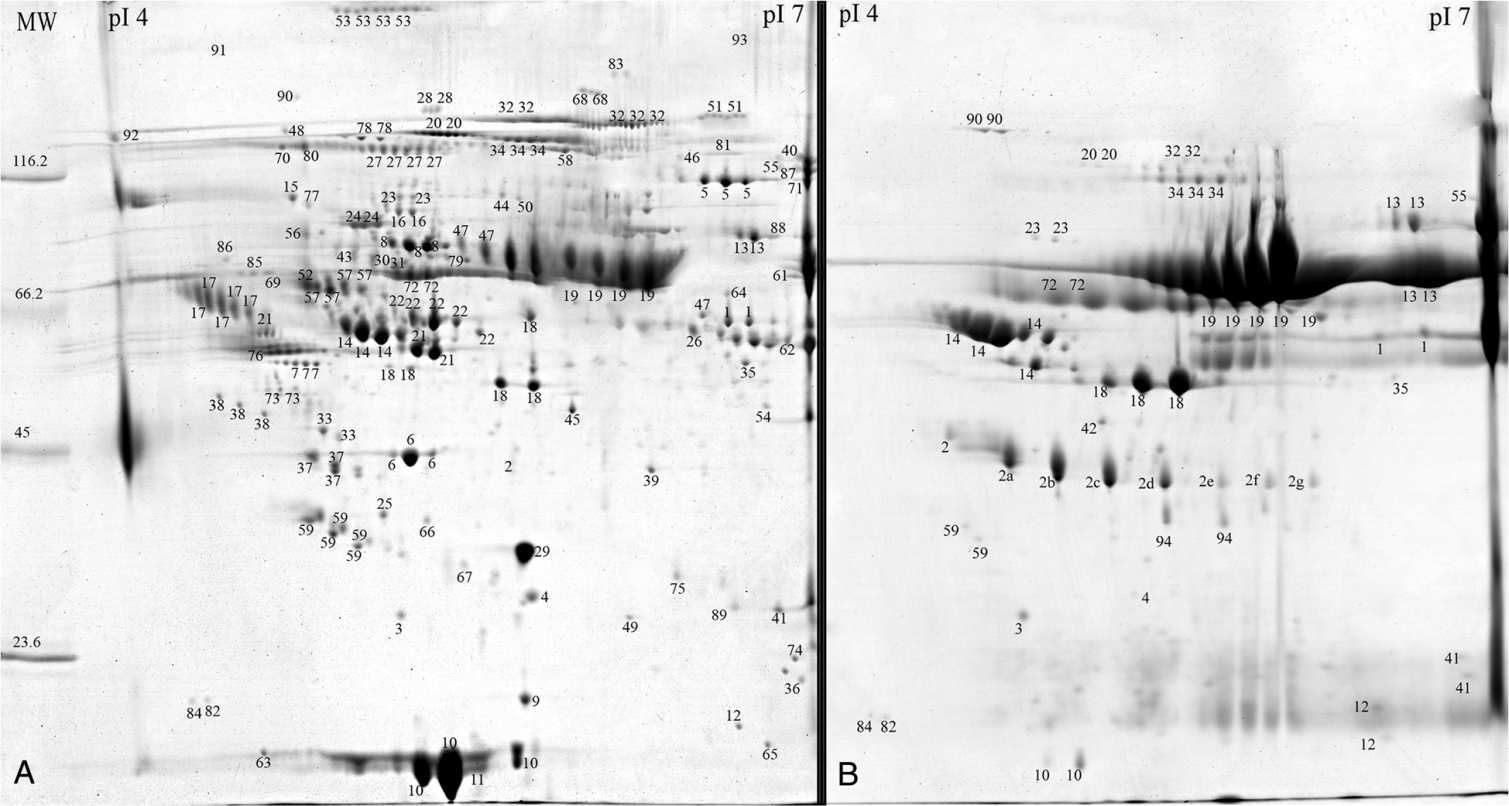
**A representative example of the 2DE analysis of the LAP (A) and HAP (B) fractions.** Plasma protein samples were depleted using the MARS Hu7 system and resolved by 2DE. IEF was performed at pH 4–7. The proteins identified by mass spectrometry are indexed by number (see Additional file 2: Table S1 and S2).

In this research depletion of seven highly abundant plasma proteins using affinity chromatography was performed and different fractions of proteins: HAPs, LAPs and LMWPs were obtained. A proteomic map obtained for the LAP fraction contained, on average, 405 ± 72 spots in all experimental groups (Figure 1A). Among them, 94 unique proteins were identified (Figure 1A, Additional file 2: Table S1). However, some amounts of the seven depleted proteins, especially albumin, were still detected (Figure 1A). The 2DE analysis of the HAP fractions resulted in dominant albumin spots and six smaller spots corresponding to the other proteins removed from blood plasma with the MARS system. In addition, tens additional protein spots were visible on the 2DE gels (Figure 1B). These spots were identified as a 15 unique proteins and are listed in the HAP fraction table (see Additional file 2: Table S2). The reproducibility of 2DE gels after MARS depletion was also assessed by scatter plotting and correlation coefficient determination based on %vol parameter. 77% of gels obtained for HAPs and LAPs had sufficient reproducibility and were approved for Image Master analysis (correlation coefficient above 0.7).

### 2DE analysis of HAPs after immunoaffinity depletion

The HAP fraction contained seven targeted proteins and 15 non-targeted proteins (Figure 1B; Additional file 2: Table S2). Comparative proteomic analyses of the HAP fraction allowed for the identification of 3 proteins with differential accumulation levels: HP, FGG and α-1 m I (Table 2A, Table 3). The relative abundances of HP and FGG were increased in the plasma of patients with CKD and CVD compared with the HV group. The highest concentrations of both proteins were detected in CKD3-4 patients (fold changes of 3.7 and 3.95, respectively; *p* value < 0.0001), then in patients with CKD1-2 (fold changes of 3.5 and 3.6; *p* value < 0.0001) and in patients with CKD5 (fold change 2.2 and 2.8; *p* value < 0.0001) compared with HV. The relative abundances of HP and FGG in CVD plasma samples were 1.92 and 1.97 times higher (*p* value < 0.0001), respectively, compared with HV samples (Table 2A).

**Table 2:** **Comparison between abundance of proteins differentially expressed between HV and CKD/CVD groups**

**A**	**Fold change-HAP**			
Protein	CKD1-2	CKD3-4	CKD5	CVD
alpha-1 m I	1.25	**2.41** ^**^	**2.63** ^**^	**1.98** ^*^
HP sum	**3.5** ^**^	**3.7** ^**^	**2.2** ^**^	**1.92** ^**^
FGG sum	**3.6** ^**^	**3.95** ^**^	**2.81** ^**^	**1.97** ^**^
**B**	**Fold change-LAP**			
Protein	CKD1-2	CKD3-4	CKD5	CVD
alpha-1 m II	**1.71** ^**^	**3.15** ^**^	**4.05** ^**^	1.50
APOA4	**1.69** ^**^	**2.18** ^**^	**2.3** ^**^	**−1.79** ^**^
APOA1	**−1.31** ^**^	**−1.36** ^**^	**−1.19** ^*^	**- 1.81** ^**^
APOB	**1.86** ^**^	**3.29** ^**^	**2.85** ^**^	**2.08** ^**^
ITIH4	**1.16** ^**^	**1.25** ^**^	**1.28** ^**^	**1.6** ^**^
TTR	1.17	1.23	**1.79** ^**^	1.17
A2M	1.03	**1.72** ^**^	**1.69** ^**^	−1.05
CFB	**1.43** ^**^	**1.48** ^**^	**1.41** ^**^	1.03
**C**	**Fold change- SRM**			
Protein	CKD1-2	CKD3-4	CKD5	CVD
alpha-1 m	**2.63** ^**^	**6.93** ^**^	**11.82** ^**^	**3.17** ^**^
APOA4	**1.52** ^**^	**2.39** ^**^	**1.71** ^**^	**−2.25** ^**^
HP	**5.6** ^**^	**17.45** ^**^	**8.19** ^**^	**3.80** ^**^
FGG	**6.87** ^**^	**7.80** ^**^	**7.81** ^**^	**2.97** ^**^
APOA1	**−1.89** ^**^	**−2.39** ^**^	**−1.99** ^**^	**- 2.61** ^**^
APOB	**2.61** ^**^	**4.21** ^**^	**3.23** ^**^	**2.81** ^**^
ITIH4	**1.52** ^**^	**1.55** ^**^	**1.51** ^**^	**1.97** ^**^
TTR	**2.0** ^**^	**1.89** ^**^	**2.90** ^**^	**1.80** ^**^
A2M	1.52	**1.92** ^**^	**2.09** ^**^	1.57
CFB	**1.78** ^**^	**1.87** ^**^	**1.54** ^**^	**1.51** ^**^

Table presented fold changes obtained for differentially expressed proteins in the HAP fraction (A), LAP fraction (B) and in SRM analysis (C). Fold changes were calculated against the HV group. * - *p* value < 0.001, ** - *p* value < 0.0001. For the actual relative abundances and standard deviations see Table 3.

**Table 3:** **Short characteristic of the proteins differentially expressed in HV, CKD and CVD groups**

**Protein**	**Accession**	**P value (ANOVA)**	**Molecular function (GO)**	**Biological process (GO)**	**Relative abundance ± SD**				
					**HV**	**CKD 1-2**	**CKD 3-4**	**CKD 5**	**CVD**
α-1-microglobulin I	AMBP_Human	< 0.0001	peptidase activity; protein binding; serine-type endopeptidaseinhibitor activity	immune system process; proteolysis; regulation of catalytic activity; metabolic process	0.104 ± 0.03	0.129 ± 0.03	0.249 ± 0.08	0.273 ± 0.06	0.206 ± 0.04
α-1-microglobulin II	AMBP_ Human	< 0.0001			0.091 ± 0.02	0.157 ± 0.04	0.289 ± 0.07	0.371 ± 0.05	0.138 ± 0.03
Apolipoprotein A-IV	APOA4_ Human	< 0.0001	enzyme regulatoractivity; protein binding; lipid binding transporter activity	cell communication; regulation of catalytic activity; response to stimulus; transport; blood circulation; lipid metabolic process	1.033 ± 0.16	1.747 ± 0.17	2.260 ± 0.24	2.378 ± 0.25	0.574 ± 0.15
Apolipoprotein A-I	APOA1_ Human	< 0.0001	enzyme regulator activity; protein binding; lipid binding; transporter activity	cell communication; regulation of catalytic activity; response to stimulus; transport; blood circulation; lipid metabolic process	4.25 ± 0.17	3.244 ± 0.13	3.116 ± 0.67	3.581 ± 0.68	2.00 ± 0.17
Apolipoprotein B-100	APOB_ Human	< 0.0001	protein binding; lipid binding; transporter activity	cell communication; regulation of catalytic activity; response to stimulus; transport; blood circulation; lipid metabolic process	0.579 ± 0.33	1.080 ± 0.10	1.907 ± 0.18	1.649 ± 0.19	1.207 ± 0.19
α-2-macroglobulin	A2MG_ Human	< 0.0001	peptidase activity; cytokine activity; serine-type endopeptidase inhibitor activity	complement activation; proteolysis; cell communication; response to stimulus; regulation of catalytic activity; lipid metabolic process; platelet activation	1.163 ± 0.10	1.202 ± 0.13	1.995 ± 0.13	1.961 ± 0.14	1.104 ± 0.16
Transthyretin	TTHY_ Human	< 0.001	protein binding; transmembrane transporter activity	cell communication; transport; metabolic process; regulation of catalytic activity; response to stimulus	2.116 ± 0.50	2.487 ± 0.81	2.617 ± 0.55	3.791 ± 0.86	2.476 ± 0.85
Inter-α-trypsin inhibitor heavy chain H4	ITIH4_ Human	< 0.0001	peptidase activity; protein binding; serine-type endopeptidase 7inhibitor activity	proteolysis; immune system process; regulation of catalytic activity; response to stimulus	1.492 ± 0.18	1.735 ± 0.07	1.861 ± 0.08	1.913 ± 0.06	2.396 ± 0.11
Complement factor B	CFAB_ Human	< 0.0001	peptidase activity; cytokine activity; serine-type endopeptidase inhibitor activity	cell communication; blood coagulation; immune system process; metabolic process; regulation of catalytic activity; response to stimulus; transport; proteolysis	0.363 ± 0.07	0.520 ± 0.06	0.593 ± 0.06	0.510 ± 0.05	0.371 ± 0.06
Haptoglobin	HPT_ Human	< 0.0001	serine-type peptidase activity; calcium ion binding; calmodulin binding; calcium-dependent phospholipid binding	gamete generation; immune system process; proteolysis; cell communication; blood circulation; response to stress; blood coagulation; metabolic process	1.073 ± 0.57	3.756 ± 0.59	3.970 ± 0.25	2.361 ± 0.65	2.060 ± 0.62
Fibrinogen γ	FIBG_ Human	< 0.0001	receptor binding	cell communication; cell-matrix adhesion; cell-cell adhesion; blood coagulation;	1.677 ± 0.63	6.039 ± 0.79	6.626 ± 1.13	4.714 ± 0.79	3.304 ± 0.72
Complement factor C4A	CO4A_Human	< 0.0001	peptidase activity; cytokine activity; serine-type endopeptidase inhibitor activity	immune system process; proteolysis; cell communication; response to stimulus; regulation of catalytic activity	5.98 ± 0.81	36.33 ± 4.61		53.98 ± 3.89	36.07 ± 0.63
Coiled-coil domain-containing protein 167	CC167_HUMAN	< 0.0001	No hit	No hit	2.72 ± 0.52	2.16 ± 0.07		6.2 ± 0.98	2.25 ± 0.39
Putative uncharacterized protein LOC100129027	YK026_HUMAN	< 0.0001	No hit	No hit	0.91 ± 0.09	1.46 ± 0.60		3.38 ± 0.36	1.48 ± 0.13

The spots corresponding to these proteins showed at least a 1.6-fold increase or reduction of relative abundance. Molecular functions and biological processes for all proteins were defined using GO annotations. Relative accumulation of proteins was calculated on the basis of the average protein abundance for all samples of each group. SD means the standard deviation of protein abundance of one certain spot.

The third differentially expressed protein was α-1 m I, fragment of preprotein AMBP. The relative abundance of α-1 m was increased in the plasma of CKD and CVD patients when compared with HVs. The highest concentration of α-1 m was detected in CKD5 patients (fold change 2.6; *p* value < 0.0001), then in CKD3-4 patients (fold change 2.4; *p* value < 0.0001). The relative abundance of this protein in CVD plasma samples was 1.98 (*p* value < 0.001) times higher compared with HV samples (Table 2A).

### 2DE analysis of LAPs after immunoaffinity depletion

**Figure 2:**
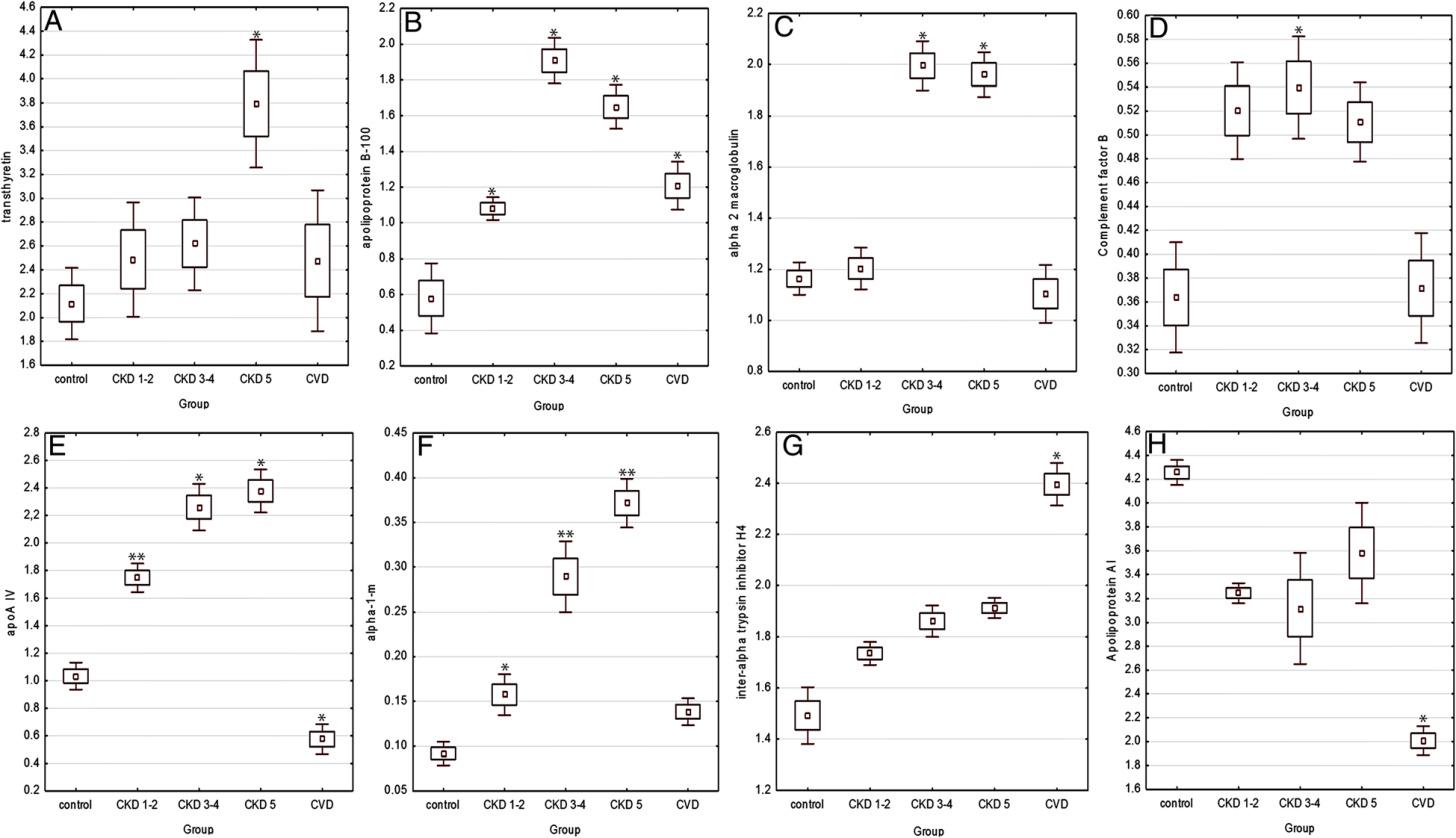
**Relative abundance of proteins differentially expressed in HV, CKD and CVD groups.** Relative accumulation (%vol) of TTR **(A)**, ITIH4 **(B)**, APOB **(C)**, A2M **(D)**, CFB **(E)**, APOA4 **(F)**, α-1 m II **(G)** and APOA1 **(H)** in HV, CKD1-2, CKD3-4 and CVD plasma samples. * - *p* value < 0.001, ** - *p* value < 0.0001. *P* values refer only to the comparison with HV group. For the other *p* values see Additional file 3.

Analysis of the LAP fractions revealed nine differentially expressed proteins: inter-alpha trypsin inhibitor H4 (ITIH4), transthyretin (TTR), apolipoprotein A1 (APOA1), apolipoprotein B100 (APOB), alpha-2-macroglobulin (A2M), apolipoprotein A-IV (APOA4), the second isoform of α-1 m protein (α-1 m II), kininogen (KIN) and complement factor C4B (CFB) (Table 2B, Table 3; Figure 2; Additional file 3).

### Comparison between HVs and CKD patients as well as inside of CKD groups

The relative abundance of TTR was increased in the plasma of CKD5 patients (fold change 1.79; *p* value < 0.0001) compared with HVs (Table 2B, Figure 2A).

APOB was up-regulated in all CKD patients compared with HVs with *p* value < 0.0001 (Table 2B, Figure 2B). The highest level of APOB was detected in CKD3-4 patients (fold change 3.29). The accumulation of this protein was 2.85 times higher in CKD5 and 1.86 times higher in CKD1-2 patients compared with HVs. Furthermore, the relative abundance of this protein was 1.76 times higher in CKD3-4 compared with CKD1-2 patients.

The relative amount of A2M was increased in the plasma of CKD3-4 and CKD5 patients, compared with HVs (Table 2B, Figure 2C). Accumulation of this protein was 1.72 times higher in the plasma of CKD3-4 patients and 1.69 times higher in the plasma of CKD5 patients compared with HVs. Significant differences in accumulation were also obtained for comparisons between CKD1-2 and CKD5 patients (fold change 1.63) as well as between CKD1-2 and CKD3-4 patients (fold change 1.65). All these results for A2M were obtained with *p* value < 0.0001.

An increase in CFB accumulation was observed only between CKD3-4 patients and HVs (fold change 1; *p* value < 0.0001) (Table 2B, Figure 2D).

The relative abundances of APOA4 and α-1 m II were increased in the plasma of CKD patients compared with HVs (Table 2B, Figure 2EF). The accumulations of these proteins were 1.72 (*p* value < 0.001) and 1.69 (*p* value < 0.0001) times higher in CKD1-2 patients for α-1 m II and APOA4, respectively, compared with HVs. The highest concentrations of both proteins were detected in CKD5 patients (fold changes of 4.07 and 2.3, respectively), and then in CKD3-4 patients (fold changes of 3.17 and 2.18, respectively). Correlation analysis of abundance of this protein in HV and all CKD patients showed correlation coefficient 0.904 (*p* < 0.001). Therefore we conclude that this increase was proportional to the level of CKD progression (Figure 2F). The relative accumulation of α-1 m II was 2.36 and 1.93 times lower in CKD1-2 compared with CKD5 and CKD3-4, respectively.

### Comparison between HVs and CVD patients

The accumulation levels of APOB and ITIH4 were up-regulated in CVD patients compared with HVs (fold changes of 2.08 and 1.6, respectively; *p* value < 0.0001)) (Table 2B, Figure 2BG).

Compared with HVs, the relative abundances of APOA4 and APOA1 were decreased in the plasma of CVD patients (fold changes of −1.79 and −2.25, respectively; *p* value < 0.0001) (Table 2B, Figure 2EH).

### Comparison between CKD and CVD patients

Finally, we compared the plasma protein levels in CVD and CKD patients. The CKD5 and CVD groups differed in the accumulation of four LAPs with *p* value < 0.0001. The relative accumulations of A2M, APOA4, α-1 m II and APOA1 were always up-regulated in CKD5 patients compared with CVD patients (fold changes 1.77, 4.14, 2.68 and 1.79, respectively) (Table 2B, Figure 2CEFH).

A comparison of the CVD and CKD3-4 plasma proteomic profiles revealed significant differences (*p* value < 0.0001) of A2M, APOA4 and α-1 m II accumulation. The relative abundances of these proteins were up-regulated in CKD3-4 compared with CVD patients, (fold changes, 1.81, 3.93 and 2.09 for A2M, APOA4 and α-1 m II, respectively) (Table 2B, Figure 2CEF).

CKD1-2 and CVD groups differed only in the accumulation of APOA4 and APOA1. Relative abundance of these proteins were 3.04 and 1.62 times higher in CKD1-2 patients compared with CVD (*p* value < 0.0001) (Table 2B, Figure 2EH; Table 2; Additional file 3).

### Analysis of LMWPs

**Figure 3:**
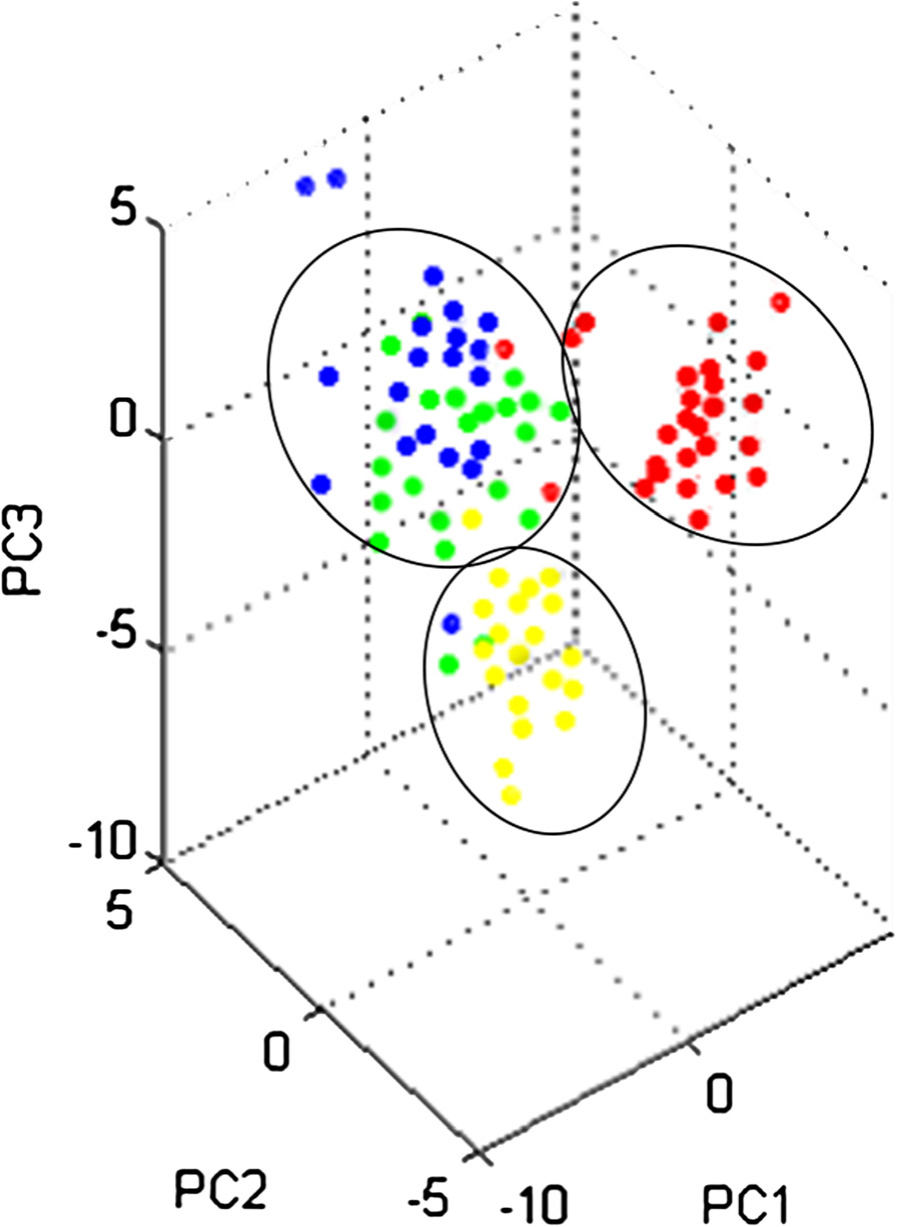
**PCA of the LMWP fraction.** PCA of the LMWP fraction obtained from the plasma of HV (yellow), CVD (blue) CKD1-2 (green) and CKD5 (red) patients. Calculations were performed with ClinProTools 2.2.

To obtain the fraction of LMWPs, individual plasma samples depleted with MARS columns were centrifuged using Agilent ultra-filters with a 5-kDa cut-off. The obtained fractions were analyzed without electrophoretic separation and without trypsin digestion in MS mode in the m/z range of 2,000-12,000 Da to find differentially expressed proteins/peptides. The obtained spectra were analyzed with ClinProTools 2.2. Thirty six differentially expressed molecules were revealed, with a threshold greater than 2 and *p* values below 0.01. PCA showed that these molecules differentiated among the HV group and CKD5 and CKD1-2/CVD patients. For these last groups, a high level of similarity was revealed in this analysis (Figure 3).

Differentially expressed LMWPs were identified in LIFT MS/MS experiments. Four components were successfully identified: transmembrane and coiled-coil domain-containing protein (C6orf129), putative uncharacterized protein and two fragments for complement factor 4 (C4A13). For putative uncharacterized protein, a Blast analysis was performed to find any functional domains. Unfortunately, this analysis revealed a lack of similarity to any known proteins. Abundance of C4A13 was up-regulated in all CKD/CVD patients compared to HV (fold changes 6.08, 6.03 and 9.03 for CKD1-2, CVD and CKD5, respectively) (Table 3). A list of the peptides/proteins of the LMWP fraction is provided in Additional file 2: Table S3.

### Validation of altered proteins by SRM analysis

**Figure 4:**
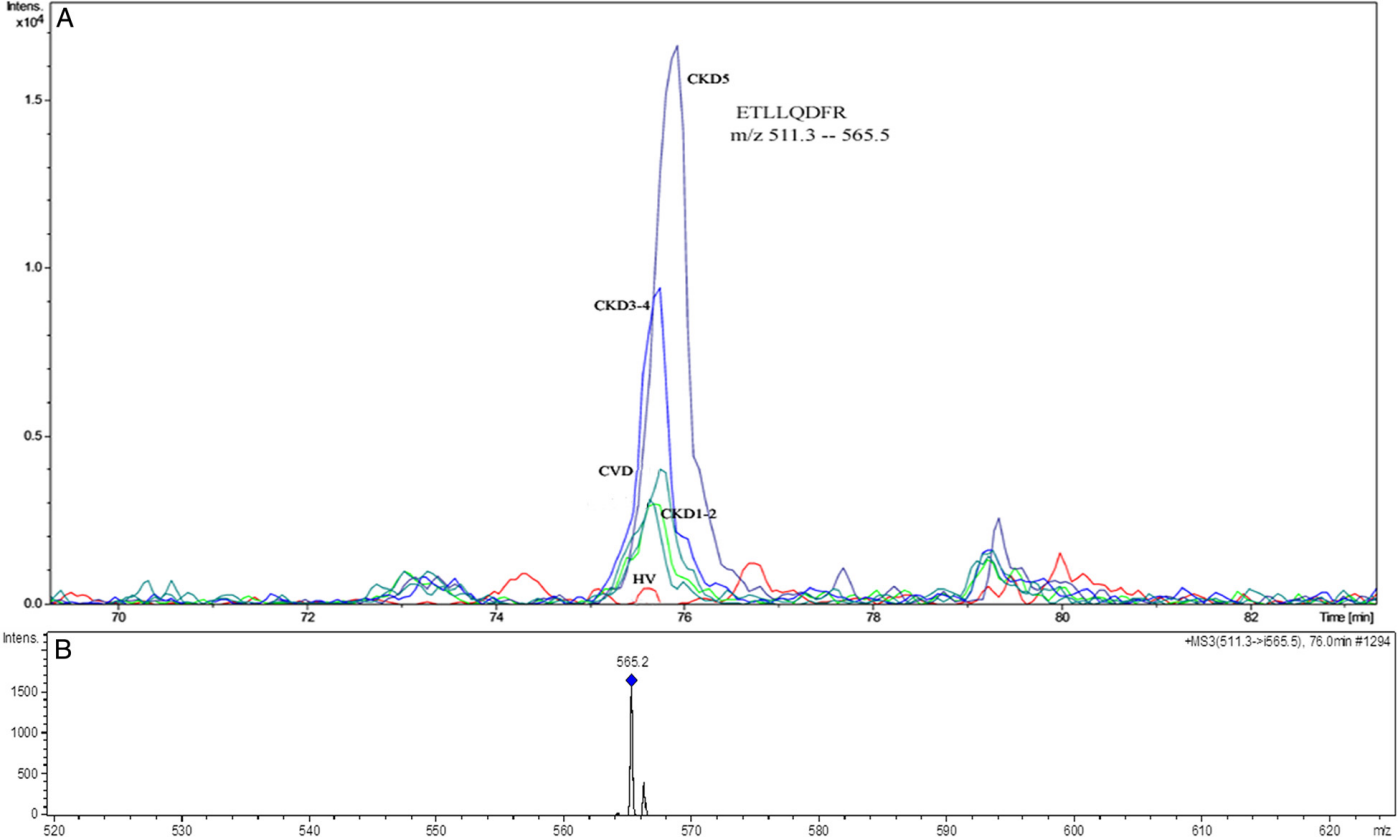
**An example of the SRM analysis of α-1 m. A**. The extracted ion chromatograms of SRM transitions of the ETLLQDFR peptide of α-1 m in HV, CKD1-2, CKD3-4, CKD5 and CVD plasma. **B**. The MS/MS spectrum of the transition 511 - > 565.5 m/z (parent mass - > fragment mass).

**Figure 5:**
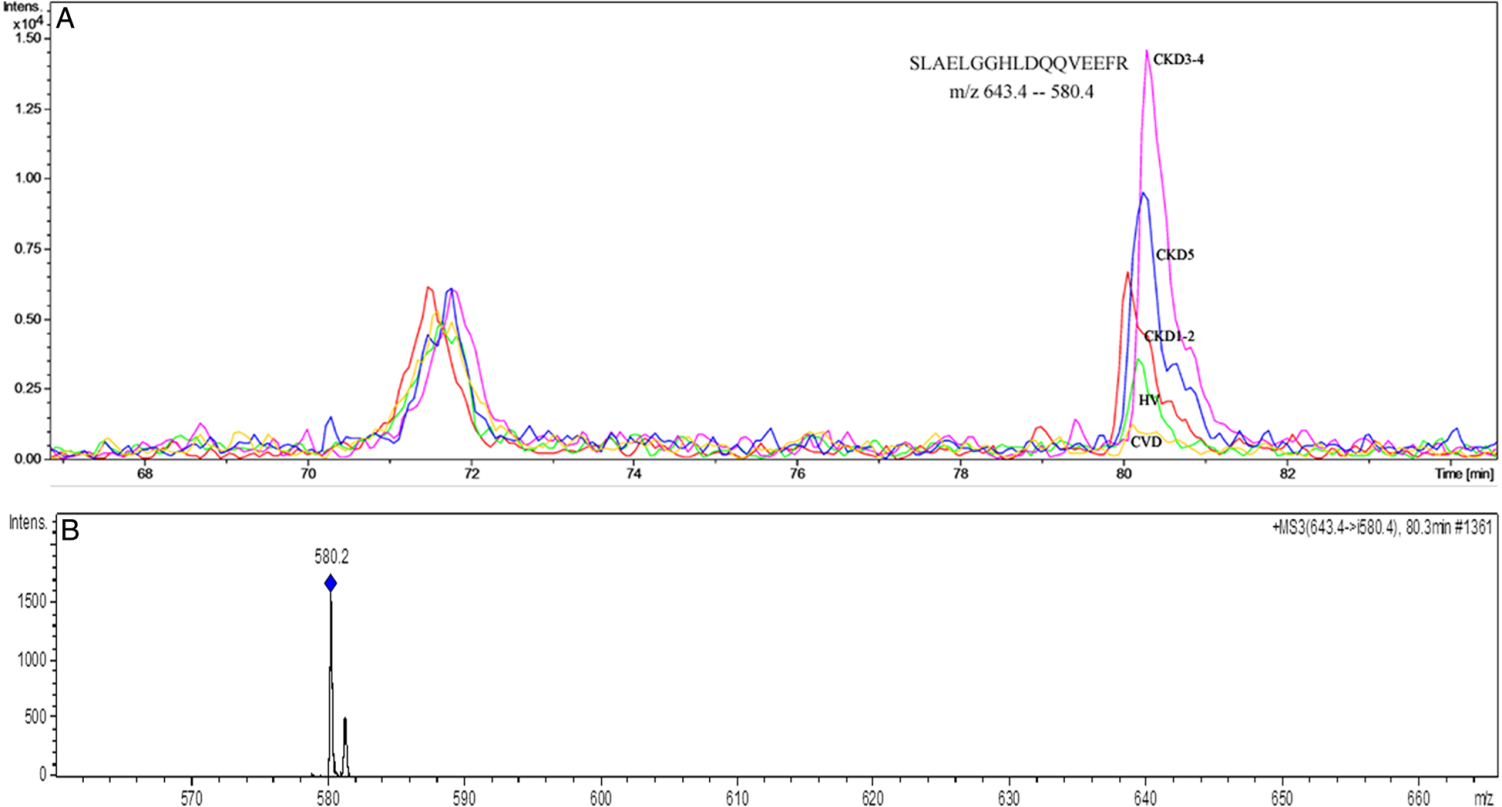
**An example of the SRM analysis of APOA4. A**. The extracted ion chromatograms of the SRM transitions of the SLAELGGHLDQQVEEFR peptide of APOA4 in HV, CKD1-2, CKD3-4 and CVD plasma. **B**. The MS/MS spectrum of the transition 643.4 - > 580.4 m/z (parent mass - > fragment mass).

To confirmation of up- or down-regulation of proteins identified in 2DE/MS analysis, SRM experiments were performed. We first optimized the SRM method using nanoLC-ESI-IT MS^n^. This technique was then used to monitor the amounts of identified proteins in samples from each analyzed group. Using the SRM approach, we successfully validated the differential accumulation of ten proteins from the HAP and LAP fractions: α-1 m, APOA4, A2M, APOA1, CFB, ITIH4, TTR, FGG, HP and APOB and one protein identified in LMWP fraction (C4A13). For these proteins, we were able to obtain specific and “clean” transitions. SRM analyses confirmed the results obtained using 2DE for all proteins except one - KIN. Therefore, this protein was excluded from the list of differentially expressed proteins and from subsequent analysis. The 10 differentially expressed proteins that were successfully validated retained the same accumulation trends as observed in 2DE analysis (Table 2, Figure 2). For some proteins insignificant in the 2DE analysis, the SRM data showed statistical significance. For TTR, the SRM analysis showed more meaningful differences in the relative abundance compared with the 2DE analysis (i.e. for comparison between the CKD3-4 and HV groups, fold change for SRM was 1.89, instead of 1.23 for 2DE analysis). Differences in accumulation between HVs and CKD1-2 or CKD3-4 patients were more visible for CFB as well (respective fold changes of 1.78 and 1.87 instead of 1.43 and 1.48 in the 2DE analysis) (Table 2B). Although the magnitude of the relative ratios of the validated proteins differed between techniques used, the direction of change in expression for the validated protein spots was consistent between 2DE and SRM analyses. It should be noted that differences in the accumulation of particular proteins were always more pronounced in SRM analysis compared with 2DE analysis. Examples of the SRM analysis for α-1 m and APOA4 peptides are shown in Figures 4 and 5.

### Identification of functional networks associated with CKD and CVD

**Figure 6:**
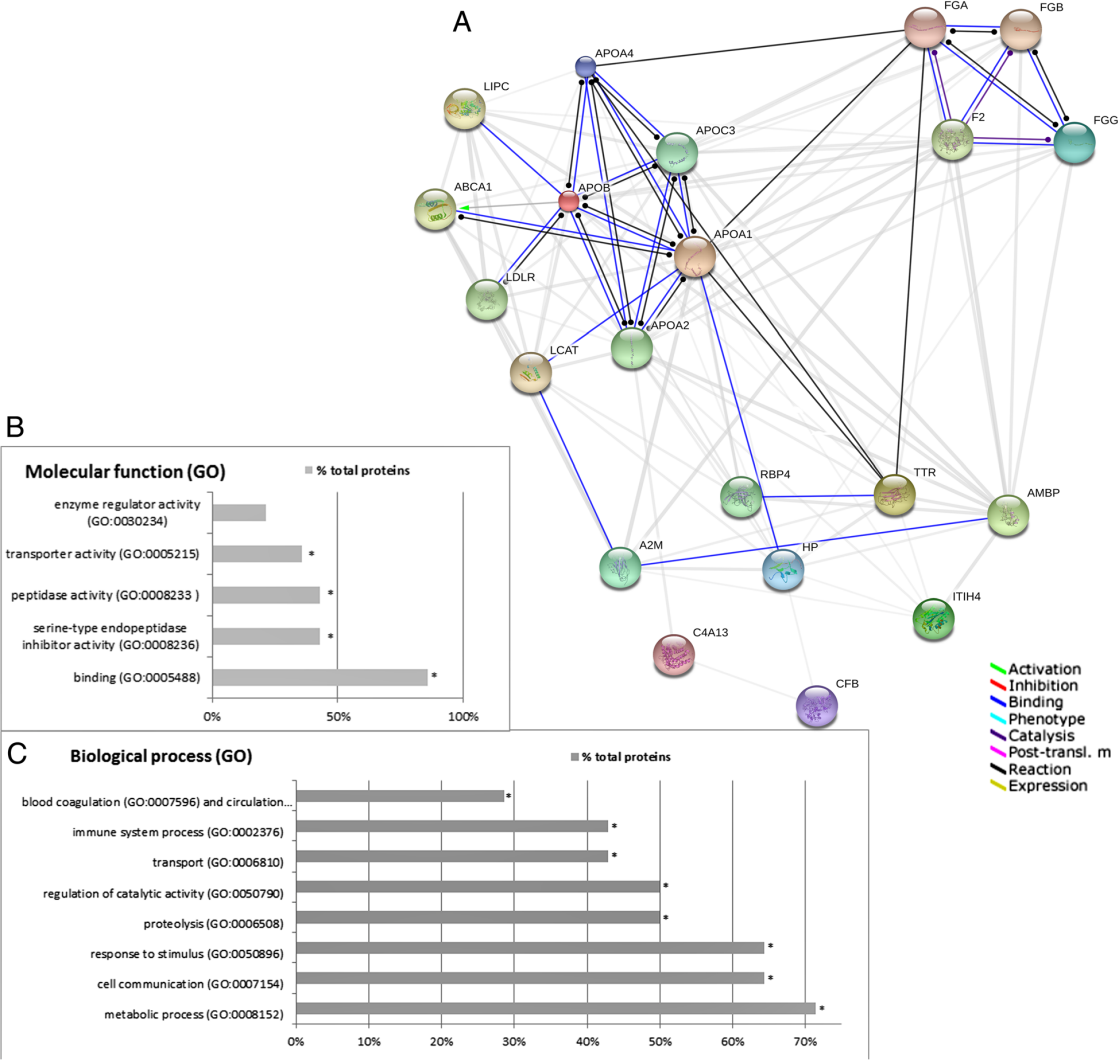
**Bioinformatic analysis of the differentially expressed proteins. A**. Predicted interaction network of the differentially expressed proteins and their putative functional partners. The proteins were analyzed using the STRING database 9.0. Nodes represent proteins. The predicted functional interaction networks are shown in the action view where the different associations are represented by differently colored lines. Each interaction between nodes is supported by the literature. APOE - apolipoprotein E; HP - haptoglobin; TTR – transthyretin; C4A13 -complement C4A; AMBP - α-1 m; ITIH4 - inter alpha-trypsin inhibitor H4; FGG – Fb gamma; APOA4 – apolipoprotein A4; C6orf127 - transmembrane and coiled-coil domain-containing protein, FGA - fibrinogen alpha chain, FGB - fibrinogen beta chain, LCAT- lecithin-cholesterol acyltransferase, LIPC - lipase, hepatic, ABCA1 - ATP-binding cassette, sub-family A (ABC1), F2 - coagulation factor II (thrombin), LDLR - low density lipoprotein receptor, APOA2 - apolipoprotein A-II, RBP4 – retinol-binding protein 4, APOC3 - apolipoprotein C-III. **B**, **C**. Classification of the identified differentially expressed proteins in molecular function **(B)** and biological processes **(C)** on the basis of gene ontology (GO) annotations. * indicate processes with *p* value below 0.05.

To gain knowledge about the molecular mechanisms of atherosclerosis that distinguish between patients with CKD and CVD we mapped protein identifiers from the source databases to their corresponding identifiers in the STRING database. As a result a network using putative protein-protein association data was created. In order to perceive the distribution of the differentially expressed proteins within important molecular functions and biological processes, clustering of differential proteins based on the Gene Ontology (GO) Consortium [14] annotations was created. STRING reveals three functional nodes: blood coagulation proteins (FGG, A2M); acute phase proteins and proteins involved either directly or indirectly in immune reactions (α-1 m, A2M, ITIH4, TTR, HP, C4A13 and CFB) as well as proteins that participate in the binding and catabolism of lipoprotein particles (APOB, APOA4, APOA1, and A2M); (Figure 6A). The most of putative connection indicate that main functions of differential proteins are binding (blue lines) and catalysis (black lines). These data are consistent with results obtained from Gene Ontology analysis. Above 85% of identified differential proteins are involved in binding function mostly in protein and lipid binding (Figure 6B). Also functions related with enzyme activity are dominated. GO analysis of biological processes shows that majority identified differential proteins are involved in metabolic processes, mainly lipid metabolic processes and cell communication (Figure 6C). Moreover, GO analysis of all identified plasma proteins (not only differentiating proteins) reveals 18 proteins involved in cell communication. Half of these proteins were up- or down-regulated in plasma CKD and CVD patients. As in the case of STRING analysis blood coagulation proteins and proteins related with immune responses (response to stimulus and immune reactions) were also relative over-represented to other proteins.

## Discussion

We performed proteomic analyses of blood plasma to increase our knowledge of the mechanisms involved in the development of atherosclerosis in CKD and to evaluate the differences between CKD- and CVD-associated atherosclerosis. We focused on three different fractions of plasma proteins: HAPs, LAPs and LMWPs. As a result, 13 differentially expressed proteins and 36 differentially expressed small molecules with masses ranging from 2 to 5 kDa were identified. It is known fact that 2DE has some limitations, especially low resolution and gel-to-gel and run-to-run variability in the expression of the same protein set. For that reason only proteins with restrictive *p* value below 0.001 and validated using SRM analysis were accepted as a differentially accumulated proteins.

Among the HAP fraction, three proteins, α-1 m I, FGG and HP, revealed differential expression in the analyzed groups. In the case of α-1 m, two isoforms were identified and both displayed differential accumulations, in the HAP (α-1 m I) and LAP (α-1 m II) fractions.

One of the main drawbacks of removing abundant proteins from plasma using affinity column is the simultaneous removal of non-targeted proteins [16]. The presence of α-1 m I in the HAP fraction may be related to its non-specific binding to proteins in the HAP fraction. A broad literature search revealed that the non-targeted removal of proteins could be accomplished using affinity systems. Approximately 50% of α-1 m in human plasma forms a complex with IgA, 7% is linked to albumins and 1% is bound to prothrombin [17] or coagulation factors (reviewed in [18]). Our study suggests that at least one of the α-1 m isoforms is associated with carrier proteins and that this isoform differentiates between the analyzed groups of patients. Furthermore, in the HAP fraction, we identified 15 other non-targeted proteins (Additional file 2: Table S2). Gundry et al. described the ‘albuminome,’ which consists of 35 proteins from plasma that co-elute with albumin via the anti-HSA depletion system [19]. From our list of identified non-targeted proteins, we found 9 proteins that match the Gundry ‘albuminome’ list (Additional file 2: Table S2). Others also showed the removal of non-targeted proteins using MARS-Hu6, MARS-Hu14 and Proteoprep20 depletion systems and some of these proteins matched our data [20-22]. Albumin seems to be a main candidate for the carrier protein. However, other proteins i.e. histidine-rich glycoproteins [23], CD5 antigen-like protein [24], clusterin [25] and HP [26] may also have a significant role in non-targeted binding. Altogether, the data obtained here confirm the fact that analyses of both fractions, LAP and HAP, after affinity depletion are necessary in the search for differential proteins and potential biomarkers.

Among the LAP fraction, eight proteins were identified and validated as differentially expressed between the analyzed groups of patients: the second isoform of α-1 m (α-1 m II), TTR, ITIH4, APOA1, APOA4, A2M, APOB and CFB (Table 2B, Table 3; Figure 2). Analysis of the obtained spectra for LMWPs revealed the presence of 36 differentially expressed proteins/peptides. Unfortunately, only four compounds were successfully identified. Bioinformatics tools were used to analyze the functional interactions of the identified differentially expressed proteins and their functional partners. These proteins were integrated into biological association networks based on the STRING database, which relies on known and predicted protein interactions [13]. All the identified proteins were analyzed also for their involvement in known biological processes and their molecular function according to GO annotation [14,15]. STRING analyses revealed that the proteins identified in our study are related to at least three different processes: the blood coagulation cascade, the transport, binding and metabolism of lipoproteins, and inflammatory processes (Figure 6A). Gene Ontology analysis also strongly indicated on protein/lipid binding and transport. Almost all differential proteins were also involved in immune reactions or responses to stimulus as well as in cell communication (Figure 6B,C).

The most puzzling result from our study is high similarity between abundance of some proteins between CKD1-2 and CVD patients. Plasma from both of these groups of patients showed similar accumulations of TTR, APOB, α-1 m I, α-1 m II, A2M, C4A13, coiled-coil domain-containing protein 167 and putative uncharacterized protein LOC100129027 (Table 3; Figure 2ABCF). Also an analysis of the obtained spectra for LMWP and performed PCA revealed high similarity between CKD1-2 and CVD patients (Figure 3). Patients from the CVD and CKD1-2 groups differ considerably in the history of the cardiac events and progress of atherosclerosis. The CVD patients selected for this study had a rich history of myocardial infarction and/or coronary angioplasty. In contrast, CKD1-2 patients showed the first symptoms of hypertension or ischemic heart disease. On the other hand CKD5 and CVD groups have similarly severe atherosclerosis symptoms and a high percentage of cardiovascular events (Table 1). However, the latter groups of patients differed in the accumulation of four identified differentially expressed proteins. These proteins (A2M, APOA4, α-1 II and APOA1) were up-regulated in CKD5 patients compared with CVD patients (Figure 2CEFH). Additionally, PCA of LMWPs showed that these groups are completely distinct from each other (Figure 3). This definitely indicates that similar proteomic profile between CKD1-2 and CVD patients are not associated with severity of atherosclerosis. This finding also supports completely different proteomic profile between CVD and CKD5 patients. It may suggest that mechanism of CVD acceleration is different in initial and advanced stages of CKD. However, these relationships need to be investigated further.

It is known that both traditional as well as novel cardiovascular risk factors support atherosclerosis progression in CKD. Our results prove undoubtedly that compared to initial CKD, patients with more advanced kidney failure have increased inflammation. This finding is confirmed not only by the increased levels of serum CRP (Table 1) but also by the differential accumulation of proteins that are involved in immune reactions and act as acute phase proteins, including α-1 m, ITIH4, TTR, HP, FGG, A2M, C4A13 and CFB, which had the highest abundances in the plasma of CKD3-4 and CKD5 patients (Table 3; Figure 2). Our results show that compared with CVD patients, the plasma of CKD3-4 and CKD5 patients demonstrated an increased acceleration of inflammatory processes and were less affected by abnormalities in cholesterol transport or metabolism. ITIH4 is a known positive acute-phase response plasma protein and is synthesized mainly in the liver [27]. Proteins CFB and C4A13 are components of the complement system and are also related to inflammatory processes [28]. Little is known about changes in the accumulation of complement factors in patients with CKD and CVD. However, atherosclerosis is a chronic inflammatory disorder that is influenced by activation of the complement system, and complement activation is extensively stimulated in atherosclerotic lesions [29-31]. Despite this, level of both proteins was higher in plasma patients with advanced CKD compared to CVD patients.

Second group of the differential proteins identified in our study are molecules involved in the transport, binding and metabolism of lipoproteins. As demonstrated by our data, changes are found in the profile of proteins including APOA4, APOA1 and APOB. Undoubtedly, these proteins are altered in CVD compared to HV. Also patients with impaired renal function exhibit significant abnormalities in profile of proteins related with lipoprotein metabolism. However character of these changes is different in CKD and CVD group of patients.

APOB and APOA1 are critical for lipoprotein formation, stability and clearance [32]. APOB, which is present in VLDL, IDL, and LDL, reflects the total number of atherogenic particles and is required for their formation [33]. High levels of APOB can enhance the progression of the formation of atherosclerotic plaques [34]. In turn, as the main protein component of anti-atherogenic HDL particles, APOA1 has anti-atherogenic potential due to its central role in the reverse cholesterol transport system and in the transfer of excess cholesterol from peripheral cells back to the liver [35,36]. Our results showed that all CKD patients have an increased level of APOB compared with HVs (SRM fold change between 2.61 and 4.21). Once again, we observe a conspicuous similarity between CKD1-2 and CVD patients (%vol 1.207 ± 0.19 and 1.080 ± 0.10 for CVD and CKD1-2, respectively) (Table 3). In contrast, a decrease in the accumulation of APOA1 was observed in all groups of CKD patients compared with HVs, but huge differences were visible when comparing CKD and CVD patients (Figure 2G). This evidence highlights the limitations of traditional lipid profile measurements, especially LDL cholesterol, as markers of cardiovascular risk in persons with CKD.

In addition to APOA1, APOA4, which was also revealed in our study as an important differentiating protein, is involved in reverse cholesterol transport and thus plays an anti-atherogenic and antioxidative role [37]. Decreased levels of APOA4 in the plasma of patients with CVD were observed. In contrast, in patients with CKD, APOA4 increased proportionally with impaired renal function. The relationship between the concentrations of this protein and progression of CKD suggests that high levels of APOA4 may have an anti-atherogenic effect, only in the case of classical CVD (Figure 2E). In CKD patients association between high level of APOA4 and an anti-atherogenic result is not efficient. Also, the APOA1 abundance suggests that the relationship between anti-atherogenic factors and the risk of cardiovascular events is not functional in patients with CKD, in contrast to classical CVD. Most likely, this risk in patients with CKD is more closely related to non-traditional risk factors, such as inflammation. The similarity between APOB abundance in CKD1-2 and CVD patients also suggests that this phenomenon is particularly more pronounced in the more advanced stages of CKD. Therefore, there is no doubt that progressive CKD is accompanied by the development of specific alterations of lipoprotein metabolism but in a manner distinct from classical CVD.

The latter observation may be explained by the discoveries from large randomized controlled trials concerning the reduction in cardiovascular risk caused by statins in CKD patients [38-40]. Lipid-lowering therapy with statins was not effective in reducing the risk of cardiovascular morbidity and mortality in patients with very advanced stages of CKD, despite the significant reduction in LDL concentrations [40-44].

This treatment was only effective in patients with mild and moderate stages of CKD [38,39,45]. In another large trial, the reduction of the risk of cardiovascular events was higher (20%) in non-dialyzed subjects when compared with hemodialyzed subjects (9%) [46]. And finally, according to the latest data statins are do not recommended for the primary prevention of CVD in dialysis patients [47]. This may suggest that risk of cardiac events in advanced CKD is not directly related with abnormalities in lipids metabolism. CKD5 patients (also in our study) have decreased serum cholesterol concentrations compared with patients with mild and moderate stages of CKD. Moreover, CKD1-2 patients exhibit increased total cholesterol and LDL cholesterol when compared with CKD3-4 patients despite the fact that greater percentage of CKD1-2 patients received statins (Table 1). Negative association between cholesterol levels and mortalities in dialyzed patients were presented few times [48-50]. Thus, this reverse epidemiology may explain the worse effects of lipid-lowering therapies in patients with advanced stages of CKD.

## Conclusions

Taken together, the findings of these analyses support the concept that different mechanisms are involved in the formation of CVD- and CKD-related atherosclerosis. APOB, APOA1 and APOA4 abundance suggests that the relationship between atherogenic and anti-atherogenic factors and the risk of cardiovascular events is not functional in patients with CKD, in contrast to classical CVD. This evidence highlights the limitations of traditional lipid profile measurements, as indicators of risk of cardiac events in CKD patients. Moreover, our results also suggest that mechanism of CVD acceleration may be different in initial and advanced stages of CKD. In more advanced stages of CKD, non-traditional risk factor, inflammation, is highly pronounced. The most puzzling result seems to be similar proteomic profiles between CVD and CKD1-2 patients, groups which are considerably different in the severity of the atherosclerosis. These relationships are unclear and further research should focus on precise resolving this issue.

## Additional files

Additional file 1:
**Details of SRM analysis.** Protein-derived peptides, transitions and retention times selected from LC-MS/MS spectra for SRM verification of the differential abundance levels of TTR, ITIH4, APOB, A2M, APOA1, CFB, α-1 m, FGG, HP and APOA4. Additional file 2:
**Proteins identified in the plasma of HVs, CKD and CVD patients. Table S1.** A. Proteins identified in the LAP fraction of the plasma of CKD and CVD patients after MARS Hu7 depletion. Spots corresponding to the particular numbers are shown in Figure 1A. RMS - root mean square value indicating on deviation from predicted mass. B, C. GO distribution of CKD/CVD plasma proteome in term of biological process (A) and molecular function (B). **Table S2.** Proteins identified in the HAP fraction of the plasma of CKD and CVD patients after MARS Hu7 depletion. Spots corresponding to the particular numbers are shown in Figure 1B. RMS - root mean square value indicating on deviation from predicted mass. **Table S3.** Results of analysis of the LMWP fraction. A. An analysis of the obtained MS spectra performed with ClinProTools 2.2 revealed the presence of 36 differentially expressed molecules with *p* values below 0.01. Three components were identified by fragmentation in MS/MS mode: transmembrane and coiled-coil domain-containing protein (C6orf129), putative uncharacterized protein and complement factor 4 (C4A13). Index - number of component; Mass - molecular mass of components; DAve - difference between the maximal and the minimal average peak area/intensity of all classes; PTTA - *p* value of t-test; CKD5, CVD, HV, CKD1-2 - peak area/intensity of given component. B. MALDI-TOF/TOF fragmentation spectrum of peptide fragment NGFKSHALQLNNRQIR, representing the C4A13 protein; the y series ions reflected the amino acid sequence. Additional file 3:
**Statistical analysis of differences in accumulation of differential proteins in HV, CKD and CVD samples.** *p* values generated using Bonferroni test (Statistica v. 10.0) for multiple testing between all analyzed experimental groups after 2DE (A) and SRM (B) analysis. Differences identified as significant (*p* > 0.001) are bolded.

## Abbreviations

CKD: Chronic kidney disease
CVD: Cardiovascular disease
CKDA: Chronic kidney disease-related atherosclerosis
HV: Healthy volunteer
SRM: Selected reaction monitoring
GFR: Glomerular filtration rate
HAP: High-abundant proteins
LAP: Low-abundant proteins
LMWP: Low-molecular weight protein
HP: Haptoglobin
FGG: Fibrinogen γ
α-1 m: α-1-microglobulin
ITIH4: Inter-alpha trypsin inhibitor H4
TTR: Transthyretin
APOA1: Apolipoprotein A1
APOB: Apolipoprotein B100
A2M: Alpha-2-macroglobulin
APOA4: Apolipoprotein A-IV
KIN: Kininogen
CFB: Complement factor C4B
C6orf129: Transmembrane and coiled-coil domain-containing protein
C4A13: Complement factor C4A

## References

[1] Eckardt K-U, Coresh J, Devuyst O, Johnson RJ, Köttgen A, Levey AS (2013). Evolving importance of kidney disease: from subspecialty to global health burden. Lancet..

[2] National Kidney Foundation (NKF) Kidney Disease Outcome Quality Initiative (K/DOQI). Advisory board (2002). K/DOQI clinical practice guidelines for chronic kidney disease: evaluation, classification, and stratification. Kidney disease outcome quality initiative. Am J Kidney Dis.

[3] Briasoulis A, Bakris GL (2013). Chronic kidney disease as a coronary artery disease risk equivalent. Curr Cardiol Rep..

[4] Weiner DE, Tighiouart H, Amin MG, Stark PC, MacLeod B, Griffith JL (2004). Chronic kidney disease as a risk factor for cardiovascular disease and all-cause mortality: a pooled analysis of community based studies. J Am Soc Nephrol..

[5] Anavekar NS, McMurray JJ, Velazquez EJ, Solomon SD, Kober L, Rouleau JL (2004). Relation between renal dysfunction and cardiovascular outcomes after myocardial infarction. N Engl J Med..

[6] Anderson TJ, Grégoire J, Hegele RA, Couture P, Mancini GB, McPherson R (2013). 2012 update of the canadian cardiovascular society guidelines for the diagnosis and treatment of dyslipidemia for the prevention of cardiovascular disease in the adult. Can J Cardiol..

[7] Kalantar-Zadeh K, Block G, Humphreys MH, Kopple JD (2003). Reverse epidemiology of cardiovascular risk factors in maintenance dialysis patients. Kidney Int..

[8] Kalantar-Zadeh K, Block G, Horwich T, Fonarow GC (2004). Reverse epidemiology of conventional cardiovascular risk factors in patients with chronic heart failure. J Am Coll Cardiol..

[9] Luczak M, Formanowicz D, Pawliczak E, Wanic-Kossowska M, Wykretowicz A, Figlerowicz M (2011). Chronic kidney disease-related atherosclerosis - proteomic studies of blood plasma. Proteome Sci..

[10] Levey AS, Bosch JP, Lewis JB, Greene T, Rogers N, Roth D (1999). A more accurate method to estimate glomerular filtration rate from serum creatinine: a new prediction equation. Modification of diet in renal disease study group. Ann Intern Med.

[11] Luczak M, Kaźmierczak M, Handschuh L, Lewandowski K, Komarnicki M, Figlerowicz M (2012). Comparative proteome analysis of acute myeloid leukemia with and without maturation. J Proteomics..

[12] Candiano G, Bruschi M, Musante L, Santucci L, Ghiggeri GM, Carnemolla B (2004). Blue Silver: a very sensitive colloidal Coomassie G-250 staining for proteome analysis. Electrophoresis..

[13] Szklarczyk D, Franceschini A, Kuhn M, Simonovic M, Roth A, Minguez P, Doerks T, Stark M, Muller J, Bork P, Jensen LJ, von Mering C (2010). The STRING database in 2011: functional interaction networks of proteins, globally integrated and scored. Nucleic Acids Res..

[14] Ashburner M, Ball CA, Blake JA, Botstein D, Butler H, Cherry JM (2000). Gene ontology: tool for the unification of biology. The gene ontology consortium. Nat Genet.

[15] Mi H, Muruganujan A, Casagrande JT, Thomas PD (2013). Large-scale gene function analysis with the PANTHER classification system. Nature Protocols..

[16] Luczak M, Marczak L, Stobiecki M (2014). Optimization of plasma sample pretreatment for quantitative analysis using iTRAQ labeling and LC-MALDI-TOF/TOF. PLoS One..

[17] Akerström B, Lögdberg L, Berggard T, Osmark P, Lindqvist A (2000). Alpha(1)-Microglobulin: a yellow-brown lipocalin. Biochim Biophys Acta..

[18] Penders J, Delanghe JR (2004). Alpha 1-microglobulin: clinical laboratory aspects and applications. Clin Chim Acta..

[19] Gundry RL, Fu Q, Jelinek CA, Van Eyk JE, Cotter RJ (2007). Investigation of an albumin-enriched fraction of human serum and its albuminome. Proteomics Clin Appl..

[20] Yadav AK, Bhardwaj G, Basak T, Kumar D, Ahmad S, Priyadarshini R (2011). A systematic analysis of eluted fraction of plasma post immunoaffinity depletion: implications in biomarker discovery. PLoS One..

[21] Stempfer R, Kubicek M, Lang IM, Christa N, Gerner C (2008). Quantitative assessment of human serum high-abundance protein depletion. Electrophoresis..

[22] Tu C, Rudnick PA, Martinez MY, Cheek KL, Stein SE, Slebos RJ (2010). Depletion of abundant plasma proteins and limitations of plasma proteomics. J Proteome Res..

[23] Leung LL (1986). Interaction of histidine-rich glycoprotein with fibrinogen and fibrin. Clin Invest..

[24] Tissot JD, Schifferli JA, Hochstrasser DF, Pasquali C, Spertini F, Clement F (1994). Two-dimensional polyacrylamide gel electrophoresis analysis of cryoglobulins and identification of an IgM-associated peptide. J Immunol Methods..

[25] Jenne DE, Lowin B, Peitsch MC, Bottcher A, Schmitz G, Tschopp JJ (1991). Clusterin (complement lysis inhibitor) forms a high density lipoprotein complex with apolipoprotein A-I in human plasma. Biol Chem..

[26] Kato GJ (2009). Haptoglobin halts hemoglobin’s havoc. J Clin Invest..

[27] Song J, Patel M, Rosenzweig CN, Chan-Li Y, Sokoll LJ, Fung ET (2006). Quantification of fragments of human serum inter-alpha-trypsin inhibitor heavy chain 4 by a surface-enhanced laser desorption/ionization-based immunoassay. Clin Chem..

[28] Hertle E, Stehouwer CD, van Greevenbroek MM (2014). The complement system in human cardiometabolic disease. Mol Immunol..

[29] Hansson GK (2005). Inflammation, atherosclerosis, and coronary artery disease. N Engl J Med..

[30] Oksjoki R, Kovanen PT, Pentikäinen MO (2003). Role of complement activation in atherosclerosis. Curr Opin Lipidol..

[31] Yasojima K, Schwab C, McGeer EG, McGeer PL (2001). Generation of C-reactive protein and complement components in atherosclerotic plaques. Am J Pathol..

[32] Marcovina S, Packard CJ (2006). Measurement and meaning of apoli-poprotein AI and apolipoprotein B plasma levels. J Intern Med..

[33] Shilpasree AS, Sahukar S, Murthy J, Kumar K (2013). A study of serum apoplipoprotein A1, apolipoprotein B and lipid profile in stroke. J Clin Diagn Res..

[34] Boren J, Ekstrom U, Agren B, Nilsson-Ehle P, Innerarity TL (2001). The molecular mechanism for the genetic disorder familial defective apolipoprotein B100. J Biol Chem..

[35] Palmer AM, Murphy N, Graham A (2004). Triglyceride-rich lipoproteins inhibit cholesterol efflux to apolipoprotein (apo) A1 from human macrophage foam cells. Atherosclerosis..

[36] Landmesser U (2012). High density lipoprotein - should we raise it?. Curr Vasc Pharmacol..

[37] Kronenberg F, Stühlinger M, Trenkwalder E, Geethanjali FS, Pachinger O, von Eckardstein A (2000). Low apolipoprotein A-IV plasma concentration in men with coronary artery disease. J Am Coll Cardiol..

[38] Koren MJ, Davidson MH, Wilson DJ, Fayyad RS, Zuckerman A, Reed DP (2009). Focused atorvastatin therapy in managed-care patients with coronary heart disease and CKD. Am J Kidney Dis..

[39] Shepherd J, Kastelein JJ, Bittner V, Deedwania P, Breazna A, Dobson S (2008). TNT (Treating to New Targets) Investigators: intensive lipid lowering with atorvastatin in patients with coronary heart disease and chronic kidney disease: the TNT (Treating to New Targets) study. J Am Coll Cardiol..

[40] Fellström BC, Jardine AG, Schmieder RE, Holdaas H, Bannister K, Beutler J (2009). AURORA Study Group: rosuvastatin and cardiovascular events in patients undergoing hemodialysis. N Engl J Med..

[41] Wanner C, Krane V, Marz W, Olschewski M, Mann JF, Ruf G (2005). German Diabetes and Dialysis Study Investigators: atorvastatinin patients with type 2 diabetes mellitus undergoing hemodialysis. N Engl J Med..

[42] Strippoli GF, Craig JC (2009). Sunset for statins after AURORA?. N Engl J Med..

[43] Kassimatis TI, Konstantinopoulos PA (2009). Rosuvastatin in patients undergoing hemodialysis. N Engl J Med..

[44] Palmer SC, Navaneethan SD, Craig JC, Johnson DW, Perkovic V, Nigwekar SU (2013). HMG CoA reductase inhibitors (statins) for dialysis patients. Cochrane Database Syst Rev..

[45] Palmer SC, Craig JC, Navaneethan SD, Tonelli M, Pellegrini F, Strippoli GF (2012). Benefits and harms of statin therapy for persons with chronic kidney disease: a systematic review and meta-analysis. Ann Intern Med..

[46] Baigent C, Landray MJ, Reith C, Emberson J, Wheeler DC, Tomson C (2011). SHARP Investigators: the effects of lowering LDL cholesterol with simvastatin plus ezetimibe in patients with chronic kidney disease (Study of Heart and Renal Protection): a randomised placebo-controlled trial. Lancet..

[47] Kassimatis TI, Goldsmith DJA (2014). Statins in chronic kidney disease and kidney transplantation. Pharmacol Res..

[48] Iseki K, Yamazato M, Tozawa M, Takishita S (2002). Hypocholesterolemia is a significant predictor of death in a cohort of chronic hemodialysis patients. Kidney Int..

[49] Liu Y, Coresh J, Eustace JA, Longenecker JC, Jaar B, Fink NE (2004). Association between cholesterol level and mortality in dialysis patients: role of inflammation and malnutrition. JAMA..

[50] Kilpatrick RD, McAllister CJ, Kovesdy CP, Derose SF, Kopple JD, Kalantar-Zadeh K (2007). Association between serum lipids and survival in hemodialysis patients and impact of race. J Am Soc Nephrol..

