# Supplementary material for: Deeper insight into chronic kidney disease-related atherosclerosis: comparative proteomic studies of blood plasma using 2DE and mass spectrometry
Source: J Transl Med. 2015 Jan 27;13:20. doi: 10.1186/s12967-014-0378-8 (PMC4316657; doi:10.1186/s12967-014-0378-8)
Supplement: Additional file 3: — Statistical analysis of differences in accumulation of differential proteins in HV, CKD and CVD samples. p values generated using Bonferroni test (Statistica v. 10.0) for multiple testing between all analyzed experimental groups after 2DE (A) and SRM (B) analysis. Differences identified as significant (p > 0.001) are bolded. [file 12967_2014_378_MOESM3_ESM.doc]

| **A** |  | **Bonferroni test for all groups** | **2DE** |  |  |  |  | **B** |  | **Bonferroni test for all groups** |  |  | **SRM** |
| --- | --- | --- | --- | --- | --- | --- | --- | --- | --- | --- | --- | --- | --- |
|  |  |  |  |  |  |  |  |  |  |  |  |  |  |
| **APOA1** | HV | CKD1-2 | CKD3-4 | CKD5 | CVD |  |  | **APOA1** | HV | CKD1-2 | CKD3-4 | CKD5 | CVD |
| HV |  | **0.0000** | **0.0000** | 0.0090 | **0.0000** |  |  | HV |  | **0.0001** | **0.0000** | **0.0001** | **0.0000** |
| CKD 1-2 | **0.0000** |  | 1.0000 | 0.8169 | **0.0000** |  |  | CKD 1-2 | **0.0001** |  | 0.0054 | 0.4570 | **0.0000** |
| CKD 3-4 | **0.0000** | 1.0000 |  | 0.2840 | **0.0001** |  |  | CKD 3-4 | **0.0000** | 0.0054 |  | **0.0000** | **0.0001** |
| CKD 5 | 0.0090 | 0.8169 | 0.2840 |  | **0.0000** |  |  | CKD 5 | **0.0001** | 0.4570 | **0.0000** |  | **0.0000** |
| CVD | **0.**0000 | **0.0000** | **0.0001** | **0.0000** |  |  |  | CVD | **0.0000** | **0.0000** | **0.0001** | **0.0000** |  |
|  |  |  |  |  |  |  |  |  |  |  |  |  |  |
| **A2M** | HV | CKD1-2 | CKD3-4 | CKD5 | CVD |  |  | **A2M** | HV | CKD1-2 | CKD3-4 | CKD5 | CVD |
| HV |  | 1.0000 | **0.0000** | **0.0000** | 1.0000 |  |  | HV |  | 0.0124 | **0.0000** | **0.0000** | 0.0021 |
| CKD 1-2 | 1.0000 |  | **0.0000** | **0.0000** | 1.0000 |  |  | CKD 1-2 | 0.0124 |  | **0.0000** | **0.0000** | 1.0000 |
| CKD 3-4 | **0.0000** | **0.0000** |  | 1.0000 | **0.0000** |  |  | CKD 3-4 | **0.0000** | **0.0000** |  | 1.0000 | **0.0000** |
| CKD 5 | **0.0000** | **0.0000** | 1.0000 |  | **0.0000** |  |  | CKD 5 | **0.0000** | **0.0000** | 1.0000 |  | **0.0000** |
| CVD | 1.0000 | 1.0000 | **0.0000** | **0.0000** |  |  |  | CVD | 0.0021 | 1.0000 | **0.0000** | **0.0000** |  |
|  |  |  |  |  |  |  |  |  |  |  |  |  |  |
| **APOB** | HV | CKD1-2 | CKD3-4 | CKD5 | CVD |  |  | **APOB** | HV | CKD1-2 | CKD3-4 | CKD5 | CVD |
| HV |  | **0.0000** | **0.0000** | **0.0000** | **0.0000** |  |  | HV |  | **0.0000** | **0.0000** | **0.0000** | **0.0000** |
| CKD 1-2 | **0.0000** |  | **0.0000** | **0.0000** | 1.0000 |  |  | CKD 1-2 | **0.0000** |  | **0.0000** | **0.0000** | 1.0000 |
| CKD 3-4 | **0.0000** | **0.0000** |  | 0.1727 | **0.0000** |  |  | CKD 3-4 | **0.0000** | **0.0000** |  | 0.0145 | **0.0000** |
| CKD 5 | **0.0000** | **0.0000** | 0.1727 |  | 0.0011 |  |  | CKD 5 | **0.0000** | **0.0000** | 0.0145 |  | **0.0000** |
| CVD | **0.0000** | 1.0000 | **0.0000** | 0.0011 |  |  |  | CVD | **0.0000** | 1.0000 | **0.0000** | **0.0000** |  |
|  |  |  |  |  |  |  |  |  |  |  |  |  |  |
| **CFB** | HV | CKD1-2 | CKD3-4 | CKD5 | CVD |  |  | **CFB** | HV | CKD1-2 | CKD3-4 | CKD5 | CVD |
| HV |  | **0.0000** | **0.0000** | **0.0001** | 1.0000 |  |  | HV |  | **0.0000** | **0.0000** | **0.0000** | **0.0000** |
| CKD 1-2 | **0.0000** |  | 1.0000 | 1.0000 | **0.0002** |  |  | CKD 1-2 | **0.0000** |  | 1.0000 | 0.0214 | **0.0000** |
| CKD 3-4 | **0.0000** | 1.0000 |  | 1.0000 | **0.0001** |  |  | CKD 3-4 | **0.0000** | 1.0000 |  | 0.0347 | **0.0000** |
| CKD 5 | **0.0001** | 1.0000 | 1.0000 |  | **0.0007** |  |  | CKD 5 | **0.0000** | 0.0214 | 0.0347 |  | **0.0000** |
| CVD | 1.0000 | **0.0002** | **0.0001** | **0.0007** |  |  |  | CVD | **0.0000** | **0.0000** | **0.0000** | **0.0000** |  |
|  |  |  |  |  |  |  |  |  |  |  |  |  |  |
| **ITIH4** | HV | CKD1-2 | CKD3-4 | CKD5 | CVD |  |  | **ITIH4** | HV | CKD1-2 | CKD3-4 | CKD5 | CVD |
| HV |  | **0.0002** | **0.0000** | **0.0000** | **0.0000** |  |  | HV |  | **0.0000** | **0.0000** | **0.0000** | **0.0000** |
| CKD 1-2 | **0.0002** |  | 0.2660 | 0.0132 | **0.0000** |  |  | CKD 1-2 | **0.0000** |  | 1.0000 | **0.0000** | **0.0000** |
| CKD 3-4 | **0.0000** | 0.2660 |  | 1.0000 | **0.0000** |  |  | CKD 3-4 | **0.0000** | 1.0000 |  | **0.0000** | **0.0000** |
| CKD 5 | **0.0000** | 0.0132 | 1.0000 |  | **0.0000** |  |  | CKD 5 | **0.0000** | **0.0000** | **0.0000** |  | **0.0000** |
| CVD | **0.0000** | **0.0000** | **0.0000** | **0.0000** |  |  |  | CVD | **0.0000** | **0.0000** | **0.0000** | **0.0000** |  |
|  |  |  |  |  |  |  |  |  |  |  |  |  |  |
| **TTR** | HV | CKD1-2 | CKD3-4 | CKD5 | CVD |  |  | **TTR** | HV | CKD1-2 | CKD3-4 | CKD5 | CVD |
| HV |  | 1.0000 | 1.0000 | **0.0000** | 1.0000 |  |  | HV |  | **0.0000** | **0.0000** | **0.0000** | **0.0000** |
| CKD 1-2 | 1.0000 |  | 1.0000 | 0.0020 | 1.0000 |  |  | CKD 1-2 | **0.0000** |  | 1.0000 | **0.0000** | 0.0139 |
| CKD 3-4 | 1.0000 | 1.0000 |  | 0.0157 | 1.0000 |  |  | CKD 3-4 | **0.0000** | 1.0000 |  | **0.0000** | 1.0000 |
| CKD 5 | **0.0000** | 0.0020 | 0.0157 |  | 0.0047 |  |  | CKD 5 | **0.0000** | **0.0000** | **0.0000** |  | **0.0000** |
| CVD | 1.0000 | 1.0000 | 1.0000 | 0.0047 |  |  |  | CVD | **0.0000** | 0.0139 | 1.0000 | **0.0000** |  |
|  |  |  |  |  |  |  |  |  |  |  |  |  |  |
| **APOA4** | HV | CKD1-2 | CKD3-4 | CKD5 | CVD |  |  | **APOA4** | HV | CKD1-2 | CKD3-4 | CKD5 | CVD |
| HV |  | **0.0000** | **0.0000** | **0.0000** | **0.0001** |  |  | HV |  | **0.0000** | **0.0000** | **0.0000** | **0.0000** |
| CKD 1-2 | **0.0000** |  | **0.0000** | **0.0000** | **0.0000** |  |  | CKD 1-2 | **0.0000** |  | **0.0000** | **0.0000** | **0.0000** |
| CKD 3-4 | **0.0000** | **0.0000** |  | 1.0000 | **0.0000** |  |  | CKD 3-4 | **0.0000** | **0.0000** |  | **0.0000** | **0.0000** |
| CKD 5 | **0.0000** | **0.0000** | 1.0000 |  | **0.0000** |  |  | CKD 5 | **0.0000** | **0.0000** | **0.0000** |  | **0.0000** |
| CVD | **0.0001** | **0.0000** | **0.0000** | **0.0000** |  |  |  | CVD | **0.0000** | **0.0000** | **0.0000** | **0.0000** |  |
|  |  |  |  |  |  |  |  |  |  |  |  |  |  |
| **alpha-1m II** | HV | CKD1-2 | CKD3-4 | CKD5 | CVD |  |  | **alpha-1m** | HV | CKD1-2 | CKD3-4 | CKD5 | CVD |
| HV |  | **0.0009** | **0.0000** | **0.0000** | 0.1161 |  |  | HV |  | **0.0000** | **0.0000** | **0.0000** | **0.0000** |
| CKD 1-2 | **0.0009** |  | **0.0000** | **0.0000** | 1.0000 |  |  | CKD 1-2 | **0.0000** |  | **0.0000** | **0.0000** | **0.0000** |
| CKD 3-4 | **0.0000** | **0.0000** |  | **0.0002** | **0.0000** |  |  | CKD 3-4 | **0.0000** | **0.0000** |  | **0.0000** | **0.0000** |
| CKD 5 | **0.0000** | **0.0000** | **0.0002** |  | **0.0000** |  |  | CKD 5 | **0.0000** | **0.0000** | **0.0000** |  | **0.0000** |
| CVD | 0.1161 | 1.0000 | **0.0000** | **0.0000** |  |  |  | CVD | **0.0000** | **0.0000** | **0.0000** | **0.0000** |  |
|  |  |  |  |  |  |  |  |  |  |  |  |  |  |
| **alpha-1m I** | HV | CKD1-2 | CKD3-4 | CKD5 | CVD |  |  | **FGG sum** | HV | CKD1-2 | CKD3-4 | CKD5 | CVD |
| HV |  | 1.0000 | **0.0000** | **0.0000** | **0.0003** |  |  | HV |  | **0.0000** | **0.0000** | **0.0000** | **0.0000** |
| CKD 1-2 | 1.0000 |  | **0.0000** | **0.0000** | 0.1712 |  |  | CKD 1-2 | **0.0000** |  | **0.0000** | **0.0000** | **0.0000** |
| CKD 3-4 | **0.0000** | **0.0000** |  | 1.0000 | 0.0140 |  |  | CKD 3-4 | **0.0000** | **0.0000** |  | **0.0000** | **0.0000** |
| CKD 5 | **0.0000** | **0.0000** | 1.0000 |  | **0.0005** |  |  | CKD 5 | **0.0000** | **0.0000** | **0.0000** |  | **0.0000** |
| CVD | **0.0003** | 0.1712 | 0.0140 | **0.0005** |  |  |  | CVD | **0.0000** | **0.0000** | **0.0000** | **0.0000** |  |
|  |  |  |  |  |  |  |  |  |  |  |  |  |  |
| **FGG sum** | HV | CKD1-2 | CKD3-4 | CKD5 | CVD |  |  | **HP sum** | HV | CKD1-2 | CKD3-4 | CKD5 | CVD |
| HV |  | **0.0000** | **0.0000** | **0.0000** | **0.0000** |  |  | HV |  | **0.0000** | **0.0000** | **0.0000** | **0.0000** |
| CKD 1-2 | **0.0000** |  | **0.0000** | **0.0000** | **0.0000** |  |  | CKD 1-2 | **0.0000** |  | **0.0000** | **0.0000** | **0.0000** |
| CKD 3-4 | **0.0000** | **0.0000** |  | **0.0000** | **0.0000** |  |  | CKD 3-4 | **0.0000** | **0.0000** |  | **0.0000** | **0.0000** |
| CKD 5 | **0.0000** | **0.0000** | **0.0000** |  | **0.0000** |  |  | CKD 5 | **0.0000** | **0.0000** | **0.0000** |  | **0.0000** |
| CVD | **0.0000** | **0.0000** | **0.0000** | **0.0000** |  |  |  | CVD | **0.0000** | **0.0000** | **0.0000** | **0.0000** |  |
|  |  |  |  |  |  |  |  |  |  |  |  |  |  |
| **HP sum** | HV | CKD1-2 | CKD3-4 | CKD5 | CVD |  |  |  |  |  |  |  |  |
| HV |  | **0.0000** | **0.0000** | **0.0000** | **0.0000** |  |  |  |  |  |  |  |  |
| CKD 1-2 | **0.0000** |  | 0.4455 | **0.0000** | **0.0000** |  |  |  |  |  |  |  |  |
| CKD 3-4 | **0.0000** | 0.4455 |  | **0.0000** | **0.0000** |  |  |  |  |  |  |  |  |
| CKD 5 | **0.0000** | **0.0000** | **0.0000** |  | 1.0000 |  |  |  |  |  |  |  |  |
| CVD | **0.0000** | **0.0000** | **0.0000** | 1.0000 |  |  |  |  |  |  |  |  |  |
